# A Patched-Like Protein PsPTL Is Not Essential for the Growth and Response to Various Stresses in *Phytophthora sojae*

**DOI:** 10.3389/fmicb.2021.673784

**Published:** 2021-10-07

**Authors:** Zhaolin Xue, Weizhen Wang, Jinghuan Shen, Jinhui Zhang, Xitao Zhang, Xili Liu

**Affiliations:** ^1^Department of Plant Pathology, College of Plant Protection, China Agricultural University, Beijing, China; ^2^State Key Laboratory of Crop Stress Biology for Arid Areas, College of Plant Protection, Northwest A&F University, Yangling, China

**Keywords:** *Phytophthora sojae*, Patched-like protein, biological function, external stresses, exogenous sterols

## Abstract

Patched (Ptc) and Patched-related (Ptr) proteins containing sterol-sensing domains (SSD) and Patched domains are highly conserved in eukaryotes for lipid transport and metabolism. Four proteins containing predicted SSD and Patched domains were simultaneously found by searching the *Phytophthora sojae* genome database, and one of them was identified as a Patched-like (PTL) protein. Here, we investigated the biological function of *PsPTL*. The expression level of *PsPTL* was higher during mycelial and sporulation stages, compared to zoospore (ZO), cyst, and germinated-cyst stages, without significant change during infection. However, deletion of *PsPTL* using CRISPR/Cas9 had no significant effect on the growth, development, or virulence of *P. sojae*. Further investigations showed that *PsPTL* is not essential for *P. sojae* to cope with external stresses such as temperature, pH, oxidative and osmotic pressure. In addition, this gene did not appear to play an essential role in *P. sojae*’s response to exogenous sterols. The transcript levels of the other three proteins containing predicted SSD and Patched domains were also not significantly upregulated in *PsPTL* deletion transformants. Our studies demonstrated that PsPTL is not an essential protein for *P. sojae* under the tested conditions, and more in-depth research is required for revealing the potential functions of *PsPTL* under special conditions or in other signaling pathways.

## Introduction

Oomycetes are filamentous microorganisms that are evolutionarily distant from fungi, with a closer phylogenetic relationship to brown algae and diatoms (Thines and Kamoun, 2010; Wu et al., 2020). *Phytophthora sojae* is one of the top 10 oomycete pathogens in molecular plant pathology and has been developed as a model oomycete (Tyler, 2007; Kamoun et al., 2015; Zhang et al., 2020). It is worth noting that the different genera in oomycetes could be differentiated by their ability to synthesize sterols (Wang et al., 2021). *Aphanomyces* and *Saprolegnia* pathogens could synthesize sterols by themselves, with many key sterol biosynthesis enzymes in their genomes, while *Phytophthora* and *Pythium* are sterol auxotrophs due to the lack of important genes for sterol synthesis (Madoui et al., 2009; Jiang et al., 2013). Since exogenous sterols can significantly promote the vegetative growth and sporulation ability of *Phytophthora* (Langcake, 1974; Nes, 1987; Galanina and Konova, 2000; Stong et al., 2013), many proteins related to sterol sensing and transport may play major roles in the process of *Phytophthora* absorbing external sterols or during its infection, just like elicitins (Blein et al., 2002).

We paid particular attention to the family of proteins containing sterol sensing domains (SSD) composed of a cluster of five consecutive transmembrane segments (TMs), which have been reported to pertain to sterol response, synthesis, or transport in many eukaryotes (Kuwabara and Labouesse, 2002; Wangeline and Hampton, 2021). The SSD-containing proteins (SCPs) mainly include the Hedgehog (Hh) pathway components Patched (Ptc or PTCH) protein, Dispatched (Disp) protein, and Ptc-related (Ptr) protein, which is a membrane protein closely related in predicted topology and domain organization to Ptc (Bolatto et al., 2015; Choi et al., 2016; Gong et al., 2018), and also include 3-hydroxy-3-methylglutaryl-CoA reductase (HMGCR), the sterol regulatory element-binding protein (SREBP)-cleavage activating protein (SCAP), Niemann-Pick type C1 (NPC1) protein, NPC1-like 1 (NPC1L1) protein, and the enzyme 7-dehydrocholesterol reductase (DHCR7), of which the predicted SSD remains controversial (Kuwabara and Labouesse, 2002; Prabhu et al., 2016).

Patched protein is the canonical receptor of the Hh pathway, along with 12 TM domains, and it was originally cloned as one of *Drosophila*’s segment polarity genes (Hooper and Scott, 1989; Nakano et al., 1989). Afterward, a human Ptc homolog was identified as a tumor suppressor for basal-cell nevus syndrome (also referred to as Gorlin syndrome; Hahn et al., 1996; Johnson et al., 1996). It has been reported that Ptc or Ptr shuttles between the plasma membrane, vesicles-like structures, and intracellular organelles (Capdevila et al., 1994; Martin et al., 2001; Bolatto et al., 2015). The Ptc and Ptr proteins seem to function in multiple aspects, including cell growth, patterning, molting, membrane integrity, and permeability (Zugasti et al., 2005; Bolatto et al., 2015; Choi et al., 2016; Jackson et al., 2020).

The classical Hh pathway mainly includes the Hh ligand, Ptc receptor, Disp transporter, Smoothened (Smo) protein, zinc finger transcription factor Cubitus interruptus (Ci) or Glioma-associated oncogene (Gli), and many other proteins in vertebrates and *Drosophila* (Ingham and McMahon, 2001; Cohen, 2003; Kong et al., 2019). Steroids play a vital role in Hh pathway, and aberrant Hh signaling leads to a series of diseases correlate with abnormal lipid metabolism (Gu et al., 2021). Hh protein is a conserved secreted protein, and its C-terminal peptide (Hh-C) autocatalyzes to produce a mature Hh protein that contains a cholesterol moiety and a palmitate moiety, before it is secreted out of cell (Porter et al., 1995). Lipid modification is essential for long-range Hh signaling and effective modulation of signaling activity by Ptc (Lewis et al., 2001; Myers et al., 2017). Mature Hh protein relieves the inhibition of Smo by binding to Ptc, and phosphorylated Smo activates Gli to move to the nucleus to promote the transcription of Hh target genes (Finco et al., 2015; Gu et al., 2021).

While, in *Caenorhabditis elegans*, a cholesterol-synthesis-deficient organism, genes encoding homologs of key members of the Hh pathway, such as Hh, Smo, and Cos2, are absent. However, two orthologs (CePTC-1, -3) of *Drosophila melanogaster* Ptc are present in *C. elegans* (Bürglin and Kuwabara, 2006). CePTC1 plays an important role in germ-line cytokinesis (Kuwabara et al., 2000), and CePTC3 is an essential protein implicated in osmoregulation of nematode and regulates the lethality of the late embryos (Soloviev et al., 2011). In plants and fungi, although Hh related genes, the Hog domain containing genes, have been identified (Bürglin, 2008), the functions of proteins in Hh pathway are still rarely studied and not well understood. Interestingly, Hh-related genes are absent in oomycetes, presumably due to gene loss, which means that the Hh pathway is incomplete in oomycetes (Bürglin, 2008). However, SCPs were found in *Phytophthora* (Wang et al., 2021), and these proteins also contain Patched domains, which means that *Ptc* or *Ptr* homologous genes may exist in *Phytophthora*. Until now, the biological functions of SCPs in oomycetes remain unknown.

The objective of the present study was therefore to (i) analyze the structural characteristics of PsSCPs; (ii) evaluate the expression profile of *Patched-like* (*PTL*) gene; (iii) observe the subcellular localization of PTL protein; (iv) determine the role of *PTL* gene in the growth and development of *P. sojae*; (v) investigate the function of *PTL* gene on *P. sojae* in sensing external stresses and exogenous sterols; and (vi) verify whether the *PsSCP* genes have functional redundancy. It is hoped that the findings will offer theoretical reference for clarifying the function of *PTL* gene in *P. sojae* comprehensively.

## Materials and Methods

### *Phytophthora sojae* Strains, Plant Cultivars, and Culture Conditions

The reference strain of *P. sojae*, P6497, was provided by Prof. Brett M. Tyler (Oregon State University) and served as the wild-type strain for gene editing to obtain mutants in this study, of which the genome has been sequenced and annotated (Tyler et al., 2006). P6497 and all transgenic strains obtained in this study were routinely cultured on 10% V8 agar medium at 25°C in darkness (Miller, 1955; Erwin and Ribeiro, 1996). Sporangium production and ZO release of *P. sojae* were performed according to a method described by Ward et al. (1989), with a few modifications.

The susceptible soybean cultivar Japan Blue was grown in plastic pots containing vermiculite at 25°C for 5–7days in a greenhouse (27±2°C, 80% relative humidity, and in the dark) for harvesting etiolated soybean seedlings.

### Nucleic Acid Isolation From *P. sojae*

Total DNA was extracted from the mycelia (MY) of *P. sojae* strains grown on 10% V8 agar medium at 25°C for 4days using the method described by Ristaino et al. (1998), with some modifications (Miao et al., 2018). Total RNA was extracted from the frozen samples using the SV Total RNA Isolation kit (Promega, Beijing, China) and cDNA was synthesized using the PrimeScript RT reagent Kit with gDNA Eraser (Takara, Beijing, China) according to the protocols of the manufacturers. All the DNA and cDNA samples were stored at −20°C until required.

### Sequence and Phylogenetic Analyses of Putative Psscps

The *PsSCPs* genes were blasted against the JGI genome database^1^ and the FungiDB data set.^2^ The sequences of SCPs from other species were collected from the National Center for Biotechnology Information (NCBI) database.^3^ The valid accession numbers of protein sequences downloaded from different databases (JGI, FungiDB, or NCBI) have been listed in Supplementary Table S1. Hidden Markov Model was used to predict putative Ptc proteins, based on the homologous Ptc proteins reported in other species (Supplementary Table S1), with the score more than 100 as a threshold (Grewal et al., 2019, 2020). The online open reading frame (ORF) finder^4^ was carried out to predict the ORFs of PsSCPs. The *PsSCP* gene models were corrected based on the RNA-seq data (FungiDB), and the reverse transcription-PCR (RT-PCR) were carried out to verify the corrected gene models. The putative transmembrane domains of PsSCPs were predicted by using TMHMM Server v.2.0.^5^ Phylogenetic analyses of PsSCPs amino acid sequences with other SCP proteins reported in different species were performed using MEGA6^6^ according to Hall (2013), and conserved domains were detected using SMART.^7^ Multiple sequence alignment of amino acid sequences generated with Clustal W (https://www.ebi.ac.uk/Tools/msa/clustalo/; Thompson et al., 1994).

### Transcription Profile of *Patched-Like* Gene in *P. sojae*

The *PsPTL* gene transcription levels were examined by quantitative real-time PCR (qRT-PCR) in different developmental stages and during the infection process. All samples, including MY, hyphae with sporangia (SP), ZO, cystospores (CY), germinated cystospores (GCY), andetiolated soybean seedlings at 0, 1.5, 3, 6, 12, 24, and 48h after inoculation, were prepared as described in the previous study with minor modifications (Ye et al., 2011). The RNA and cDNA were obtained according to the above method and stored at −80°C until required. The primers used for this experiment were shown in Supplementary Table S2, and the *P. sojae* housekeeping genes *RPS* and *RPL13α* were served as endogenous controls (Wang et al., 2011). The qRT-PCR reactions were performed following the manufacturer’s instructions (FastSYBR Mixture, CW Biotech, Beijing, China) by using a qPCRsoft 3.4 system (qTower 2.2, Analytik Jena AG, Jena, Germany) under the following conditions: 95°C for 2min, 40cycles of 95°C for 10s, 60°C for 30s to calculate cycle threshold (Ct) values, followed by 95°C for 15s, 60°C for 1min, and then 95°C for 15s to obtain melt curves. The relative expression levels of each gene were calculated using the 2^−ΔΔCt^ method (Cheng et al., 2019). Two biological replicates, each containing three technical replicates, were carried out for each sample.

### Subcellular Localization of PTL Protein

The *eGFP* gene was fused to the C-terminus of *PsPTL* by ligation to *SpeI*/*ApaI*-digested pYF3 (Fang et al., 2017b). The primers used for sequence amplification are shown in Supplementary Table S2. The pYF3-PsPTL-eGFP construct was introduced into *P. sojae* P6497 by polyethylene glycol (PEG)-mediated protoplast transformation (Fang and Tyler, 2016), and the empty construct pYF3-eGFP was transformed into P6497 as a negative control. The living hyphae of transformants were picked from the 10% cleared V8 broth containing 50μg/mlG418 after 3days growth, which were observed by using confocal microscopy (Olympus Fluoview FV3000). Images were captured using a 60× oil objective with excitation/emission settings (in nm) 488/510~535 for green fluorescent protein (GFP).

### *PsPTL* Gene Deletion in *P. sojae*

*PsPTL* gene deletion mutants were generated using the CRISPR/Cas9 System (Fang and Tyler, 2016). According to the previous protocol (Fang and Tyler, 2016), three single guide RNA (sgRNA) listed in Supplementary Table S2 were designed and cloned into the backbone plasmid pYF2.3G-ribo-sgRNA, respectively. The *NPTII* gene ligated with two 1,000bp fragments flanking *PsPTL* gene was used as donor DNA in homology-directed repair (HDR) and infused into the basic donor plasmid pBS-SK+ using the primers listed in Supplementary Table S2 and the In-Fusion HD Cloning Kit (Clontech, Mountain View, CA, United States). All DNA fragments were amplified using TransStart® FastPfu DNA Polymerase (TransGen Biotech, Beijing, China). We transformed *P. sojae* wild-type P6497 using PEG-mediated protoplast transformation using previously described methods (Fang and Tyler, 2016; Fang et al., 2017a). Putative transformants were transferred to the 10% V8 agar medium containing 50μg/ml G418 and incubated for 3days at 25°C. For verification of the transformants, their genomic DNA was extracted and the homologous recombination events were verified by PCR using primers as shown in Supplementary Table S2. Sanger sequencing and qRT-PCR were also used to confirm that the *PsPTL* gene was cleanly replaced by *NPTII* gene and absent in the homozygous deletion transformants.

### Biological Characteristics Analysis of *P. sojae*

To determine mycelial growths of *P. sojae* wild-type (P6497) and transformants, colonies were measured after depositing a mycelial plug (5mm in diameter) on 10% V8 agar medium after 4days (Miao et al., 2018).

To analyze SP production, 10 mycelial plugs (5mm in diameter) from the edge of actively growing colonies on the V8 juice agar medium were placed into a Petri dish, containing 20ml of 10% clarified V8 broth. These plates were incubated at 25°C in the dark for 3days then the hyphae were repeatedly washed with 20ml of sterile distilled water (SDW) for five times, followed by incubation in the dark at 25°C for 4–8h, until sporangia developed. The number of sporangia was counted using the light microscope (magnification ×100).

To gauge the zoospore production of *P. sojae*, zoospores were produced as described above. After washing the mycelia five times, another 10-ml SDW was added into the Petri dishes, which were incubated in the dark at 25°C for 8–10h. The number of zoospores was counted using a hemocytometer.

To assess cyst germination, the zoospore suspensions were shaken using a vortex for 1min in order to complete cystospore encystment. The cystospore suspension was incubated at 25°C for 6h in the dark. More than 100 cystospores were examined under the microscope, which were considered as germinated if the germ tube was longer than the cystospore diameter.

To evaluate oospore production, the mycelial plug (5mm in diameter) was cultured on 10% V8 agar for 7days. Then, the number of oospores was counted using the light microscope (magnification ×10). Three duplicates of each strain were incubated in darkness at 25°C, and the experiment was repeated three times.

To investigate the pathogenicity of *P. sojae*, the etiolated seedlings of soybean cultivar Japan Blue were inoculated with mycelium plugs (5mm in diameter) on the hypocotyls, and maintained at 25°C and 80% relative humidity in the dark for 3days. Then, the lesion length was measured. Each strain was tested using at least six seedlings. All assays were repeated three times.

### Response of *PsPTL* Mutants Under Different Stress Conditions

To determine the response of *P. sojae* to temperature, the wild-type (P6497), control strain (EV), and *PsPTL* mutants were grown on Plich medium (Qiu et al., 2020) at 10, 18, 25, 30, and 37°C, respectively. Colony diameters were measured after incubation in the dark for 7days, and each treatment consisted of three replicated plates.

To determine the response of *P. sojae* to pH stress, all strains were grown on Plich medium with a pH value of 5, 6, 7, 8, and 9 at 25°C, respectively, in the dark for 7days. Colony diameters were measured, and each treatment consisted of three replicated plates.

To determine the response of *P. sojae* to osmotic stress, all strains were grown on Plich medium amended with KCl (0, 0.25, and 0.5M), as well as sorbitol (0, 0.5, and 1M) at 25°C in the dark for 7days, respectively. Colony diameters were measured, and each treatment consisted of three replicated plates.

To determine the response of *P. sojae* to oxidative stress, all strains were grown on Plich medium amended with 0, 2, and 5mM H_2_O_2_, respectively, at 25°C in the dark for 7days, respectively. Colony diameters were measured, and each treatment consisted of three replicated plates.

### Response of *PsPTL* Mutants to Exogenous β-Sitosterol

To determine the response of *P. sojae* to exogenous sterol, the wild-type (P6497), control strain (EV), and *PsPTL* mutants were grown on basal medium (Jee and Ko, 1997) amended with 0, 5, and 20μg/ml β-sitosterol (Shanghai Yuanye Bio-Technology Co., Ltd., Shanghai, China). All strains were measured after incubation in darkness at 25°C for 7days. Each treatment consisted of three replicate plates, and the experiment was repeated three times.

### Other *PsSCPs* Transcript Levels Analysis in *PsPTL* (*PsSCP3*) Mutants

All mycelial samples of *PsPTL* (*PsSCP3*) mutants were collected after incubation in darkness at 25°C for 4days on 10% V8 medium. The RNA and cDNA were obtained according to the above method and stored at −80°C until required. Transcript levels of other *PsSCPs* genes were measured by qRT-PCR. The primers used for this experiment were shown in Supplementary Table S2, and the *P. sojae* housekeeping genes *RPS* and *RPL13α* were served as endogenous controls (Wang et al., 2011). The qRT-PCR reactions were performed as described above. The relative expression levels of each gene were calculated using the 2^−ΔΔCt^ method (Cheng et al., 2019). Two biological replicates, each containing three technical replicates, were carried out for each sample.

### Statistical Analysis

All statistical analyses were conducted using DPS software version 7.05. Differences between the means were determined by the least significance difference (LSD) multiple range test at *p*=0.05.

## Results

### Sequence and Phylogenetic Analyses of Four Putative PsSCPs

A total of 59 predicted Ptc proteins (Supplementary Table S3) were found in the genome of *P. sojae* P6497 by searching for the conserved domains in JGI database,^8^ and predicted by Hidden Markov Model. However, it was found that there are positional overlaps among different genes, and only six genes are in different positions, with the protein IDs of 466,676, 318,600, 549,457, 322,952, 389,466, and 564,545, respectively (Supplementary Table S3). We further searched RNA-seq data of these genes in FungiDB, and found that 466,676 and 322,952 are individually expressed in a continuous mode. Although, 318,600 and 549,457 are annotated as two different genes, they are expressed together uninterruptedly, which is similar to 389,466 and 564,545 (Supplementary Figure S1). The prediction of ORF and full-length amplification by RT-PCR were carried out to correct the gene models, and four independent genes were identified, because 318,600 and 549,457 with their sequence interval constituted one independent gene, and 389,466 and 564,545 with their sequence interval constituted another independent gene. We artificially named these four proteins as PsSCP1-4, which referred to 466,676, 318,600 and 549,457, 322,952, and 389,466 & 564,545, and the corrected sequences were shown in the Supplementary Material. Four homologous proteins were also found in the genome of *Phytophthora capsici*,^9^ and they were denoted as PcSCP1, 2, 3, and 4, correspondingly.

**Figure 1:**
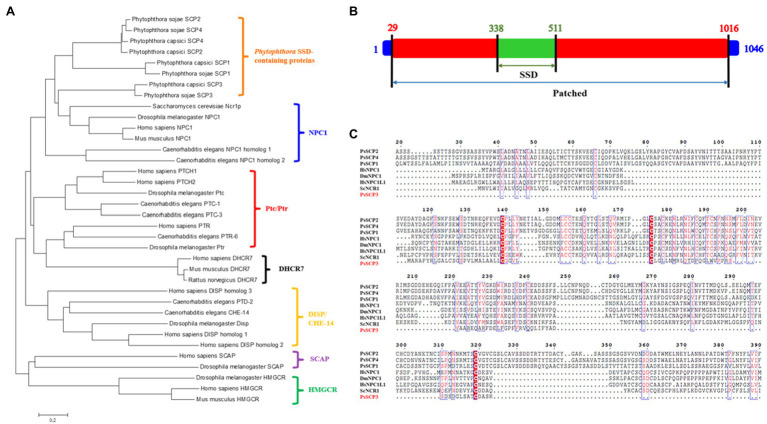
Sequence characteristics of four putative PsSCPs. **(A)** Phylogeny analysis of PsSCPs with other SSD-containing proteins (SCPs) reported in different species. **(B)** Full length of PsPTL (PsSCP3) protein and location of the sterol-sensing domain (SSD) and Patched (Ptc) domain. **(C)** Multiple sequence alignment of N-terminal amino acid sequences of PsSCPs with deduced Niemann-Pick type C (NPC) domain of NPC-related proteins from other species, generated with Clustal W (Thompson et al., 1994; Hs, *Homo sapiens*; Dm, *Drosophila melanogaster*; Sc, *Saccharomyces cerevisiae*).

The phylogenic analysis showed that PsSCPs and PcSCPs were organized into a cluster of NPC1 homologs (Figure 1A). The conserved domain prediction of PsSCPs and PcSCPs confirmed that the N-terminal NPC1 domain (NPC1-N), SSD, and Patched domains are present in SCP1, 2, and 4 (Supplementary Figure S2). However, it found that SCP3 did not contain NPC1-N domain (Figures 1B,C). The distribution and composition of conserved domains are also different between PsSCP3 and the other three proteins (Supplementary Figure S2; Figure 1B). In other species, both NPC1 and Patched (Ptc) proteins have SSD and Patched domains, while the N-terminal domain of NPC1 (NPC1-N) is only present in the homologous proteins of NPC1 (Loftus et al., 1997; Malathi et al., 2004). NPC1-N plays a vital role in binding cholesterol (Qian et al., 2020). NPC1 and Ptc proteins are involved in different signaling pathways, and their mutants showed distinct phenotypes, which may be due to the special presence of NPC1-N (Kuwabara and Labouesse, 2002). In *Phytophthora*, PsSCP1, 2, 4 display similar protein structures to other NPC1 homologs with the specific NPC1-N domain (Loftus et al., 1997; Malathi et al., 2004), and it is found that they are more closely related to NPC1 proteins in the phylogenetic analysis (Figure 1A). Because PsSCP3 does not contain the specific NPC1-N domain and similar protein structure to NPC1 homologs, and has similar domains as Ptc, it may be the PTL protein. We performed a phylogenic analysis of SCP proteins in other oomycetes, such as *Phytophthora ramorum*, *Phytophthora infestans*, *Peronospora effusa*, *Phytophthora parasitica*, and *Phytopythium vexans*. We found that PTL (SCP3) proteins were conserved in different species of oomycetes, and evolved independently into a branch that is similar to Patched protein (Supplementary Figure S3). Hence, PsSCP3 was renamed artificially as PsPTL protein, and amplified for a full length of 3,138bp, of which the open reading frame includes one intron and encodes a 1,046-amino-acid protein (Figure 1B), with 11 putative transmembrane domains. The biological function of PsPTL has been further studied in this research.

### Transcription Profile of *PsPTL*

**Figure 2:**
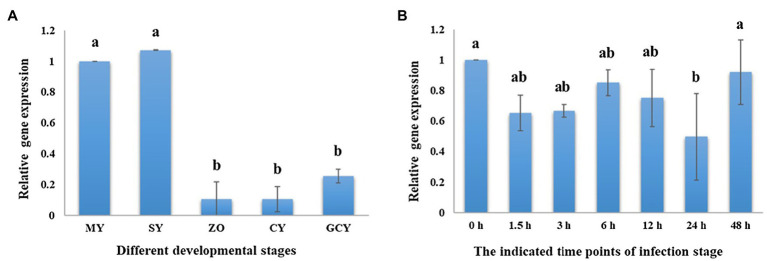
*PsPTL* gene transcription profile. **(A)** Sample tissues of *P. sojae* P6497 included mycelia (MY), hyphae with sporangia (SP), zoospores (ZO), cystospores (CY), and germinated cystospores (GCY), and the transcript levels of other samples are shown relative to MY, which was assigned a value of 1.0. **(B)** The samples of infection stages were collected for 0, 1.5, 3, 6, 12, 24, and 48h after infection of susceptible soybean cultivar Japan blue, and the transcript levels other samples are shown relative to the sample of 0h, which was assigned a value of 1.0. Columns indicate means, and bars signify SD. Different letters above the columns indicate statistically significant differences (*p*<0.05).

We found that *PsPTL* showed higher expression during the mycelial and sporulation stages, compared to the zoospore, cyst, and germinated-cyst stages (Figure 2A), indicating that *PsPTL* may play an important role in mycelial growth and sporulation. Similar transcriptional expressions of *PsPTL* were found in the mycelium (0h) and during all indicated time points of infection (1.5–48h after inoculation; Figure 2B), which suggests that *PsPTL* may not have a prominent function in the pathogenic stage of *P. sojae*.

### Subcellular Localization of PTL Protein

**Figure 3:**
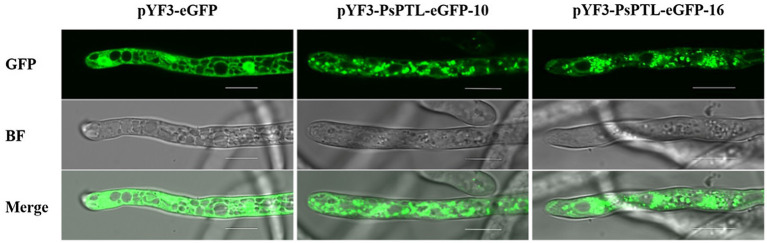
Subcellular localization of Patched-like (PTL) protein in *P. sojae* hyphae. The hyphae of transformants expressing pYF3-PsPTL-eGFP or pYF3-eGFP, respectively, were observed in green fluorescent protein (GFP) field (top), bright field (BF, middle), and merged field (bottom). Bars represent 10μm.

The eGFP-tagged PsPTL protein was observed in the hyphae of *P. sojae* by confocal microscopy (Olympus Fluoview FV3000). The green fluorescence of the transformants expressing solo eGFP distributed throughout the hyphae except some vacuoles, and the strongest fluorescence signal was in the nucleus, which has been observed in the study of Fang et al. (2017b). While, in the transformants expressing the eGFP-tagged PsPTL protein, green fluorescence mainly scattered or accumulated in the cytoplasm with a punctate or spot structure, which was different from the transformants that only expressed eGFP (Figure 3).

### Biological Characteristics of *PsPTL* Mutants

**Figure 4:**
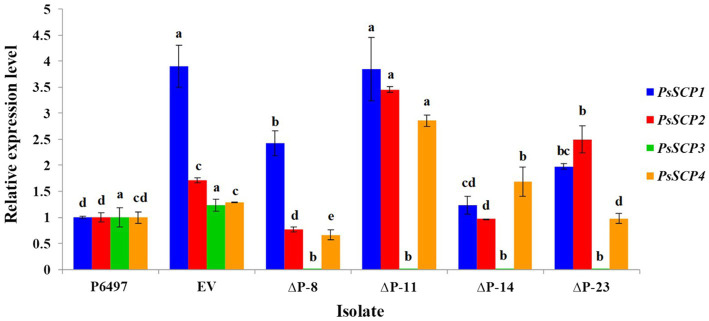
*PsSCPs* transcript levels in *PsPTL* (*PsSCP3*) mutants. The transcript levels of transformants are shown relative to wild-type P6497, which was assigned a value of 1.0. Columns indicate means, and bars show SDs. The same letters above the same color column signify statistically insignificant difference (*p*<0.05).

**Figure 5:**
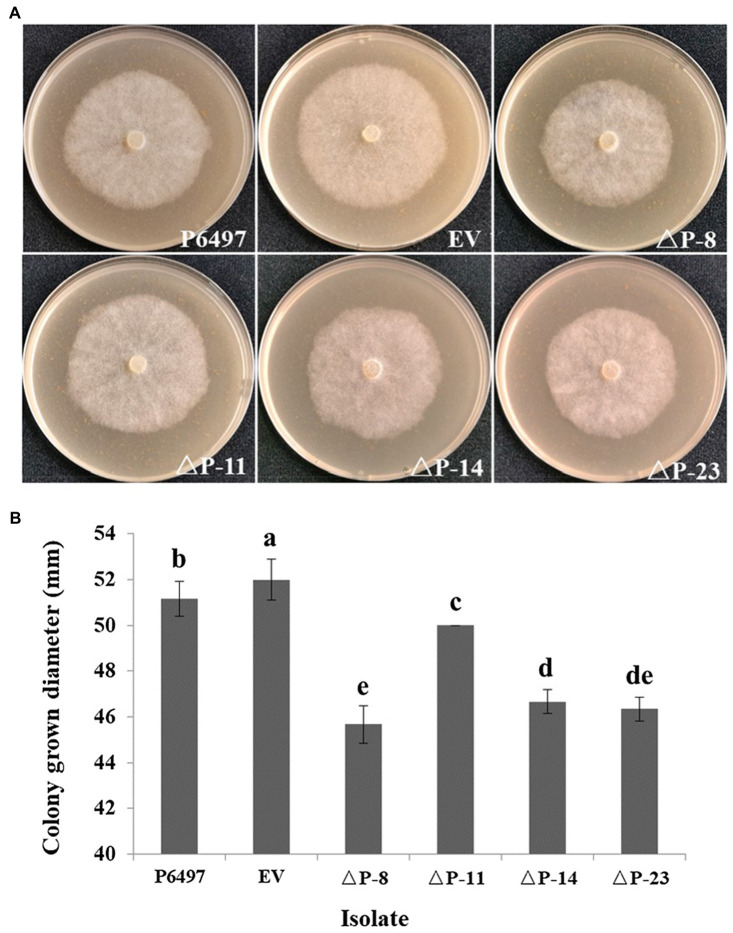
Mycelial growths of *PsPTL* mutants. **(A)** Growth characteristics after 4days on 10% V8 agar medium of the wild-type (P6497), control strain (EV), and *PsPTL* mutants (ΔP-8, 11, 14, 23). **(B)** Mycelial growth diameters after 4days on 10% V8 agar medium. Columns indicate means, and bars signify SDs. Different letters above the columns indicate statistically significant differences (*p*<0.05).

**Figure 6:**
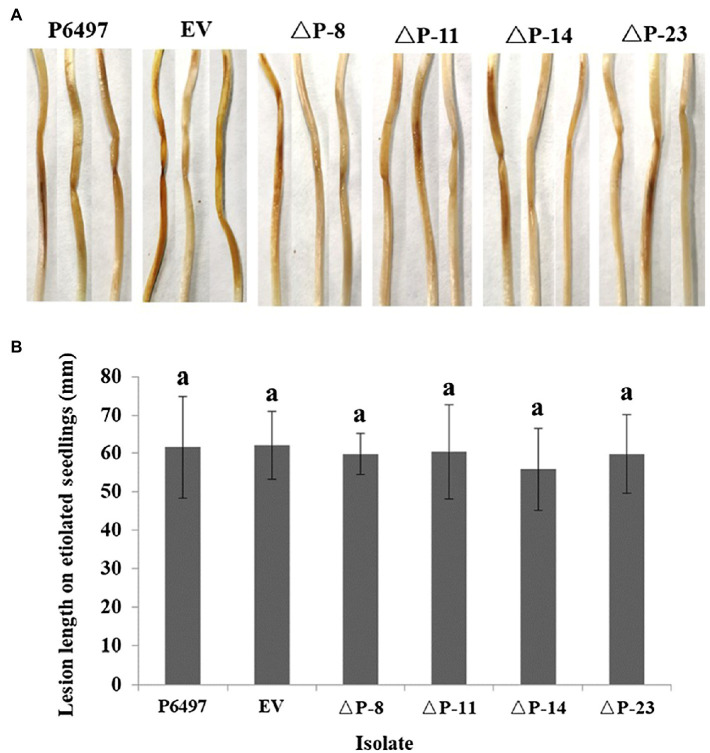
Virulence of *PsPTL* mutants. **(A)** Lesions on etiolated soybean hypocotyls. **(B)** Lesion length on etiolated soybean seedlings. Columns indicate means, and bars show SDs. The same letters above the columns signify statistically insignificant difference (*p*<0.05).

To explore the biological function of *PsPTL* in *P. sojae*, the wild-type strain P6497 had been genetically modified and four independent homozygous mutants (ΔP-8, ΔP-11, ΔP-14, and ΔP-23) were obtained and confirmed by Sanger sequencing (Supplementary Figure S4) as well as qRT-PCR (Figure 4). The transformant in which the *PsPTL* deletion was not successful was included as a negative control (EV). The biological characteristics of all *PsPTL* deletion transformants, the wild-type and EV strains, were analyzed. For mycelial growth, all transformants showed slightly slower growth rates than the wild-type strain P6497 and the negative control (Figure 5). The sporangium production and zoospore production of all mutants displayed no significant difference compared to the wild-type strain P6497 or EV transformant. It was found that the disruption of *PsPTL* did not significantly affect the cystospore germination of *P. sojae*, except for mutant ΔP-14, which had a significantly lower cystospore germination rate. In addition, mutants ΔP-8 and ΔP-11 produced a considerable number of oospores relative to P6497, whereas ΔP-14 and ΔP-23 produced fewer oospores (Table 1). In addition, no significant difference was found in virulence among all the strains (Figure 6).

**Table 1:** Asexual and sexual spores of *PsPTL* mutants in *Phytophthora sojae*.

Isolate	No. sporangia	No. zoospores	Cystospore germination (%)	Oospore production
P6497	340a^*^	33.67a	63.46a	586b
EV	195b	17.67b	63.50a	778a
ΔP-8	367a	19.00b	60.74ab	494b
ΔP-11	326ab	14.67b	48.59ab	497b
ΔP-14	189b	17.88b	39.51b	304c
ΔP-23	240ab	13.63b	42.54ab	304c

* *Different letters after the numbers indicate statistically significant differences by Fisher’s protected least significance difference (LSD) test (p<0.05)*.

### Response of *PsPTL* Mutants Under Different Stress Conditions

**Figure 7:**
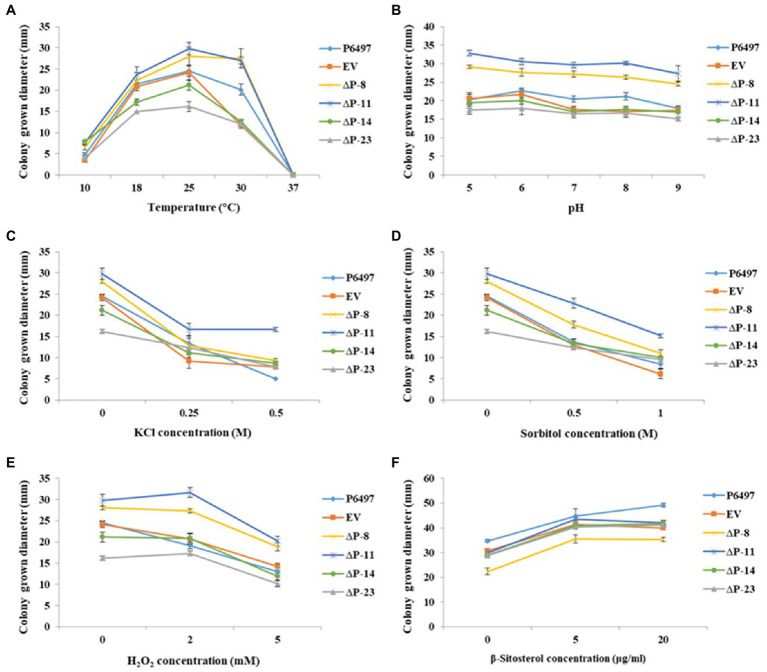
Mycelial growths of *PsPTL* mutants under different stress conditions and exogenous β-sitosterol. **(A)** Mycelial growth diameters of the wild-type (P6497), control strain (EV), and *PsPTL* mutants (ΔP-8, 11, 14, 23) on Plich medium at 10, 18, 25, 30, and 37°C, respectively; **(B)** and on Plich medium with a pH value of 5, 6, 7, 8, and 9; **(C)** with 0, 0.25, and 0.5M KCl; **(D)** with 0, 0.5, and 1M sorbitol; **(E)** with 0, 2, and 5mM H_2_O_2_, respectively. **(F)** Mycelial growth diameters of all strains on basal medium amended with 0, 5, and 20μg/ml β-sitosterol. Bars indicate SDs.

To determine whether *PsPTL* plays an essential role in coping with external stresses of *P. sojae*, *PsPTL* mutants and wild-type strain P6497 were cultured under various environmental stresses, such as temperature, pH, osmotic stress, and oxidative stress. The optimum temperature for mycelial growth of all transformants and P6497 was 25°C. Transformants ΔP-8 and ΔP-11 showed slightly faster mycelial growth rates than the wild-type P6497 and EV transformant, whereas ΔP-14 and ΔP-23 showed slower mycelial growth rates (Figure 7A). Further, all mutants did not have significant changes in response to pH conditions, oxidative stress (H_2_O_2_), and osmotic stress (KCl and sorbitol), compared to P6497 and EV (Figures 7B–E).

### Response of *PsPTL* Mutants to Exogenous β-Sitosterol

We speculated that the *PsPTL* deletion may affect the influence of exogenous sterols on *P. sojae* because of the conserved SSD domain. Therefore, mycelial growths of *PsPTL* mutants and wild-type strain P6497 were determined with exogenous β-sitosterol. However, the results showed that the growth pattern of the mutants did not change significantly compared with the wild-type or EV strain after adding different concentrations of exogenous β-sitosterol (Figure 7F), indicating that the deletion of *PsPTL* might not affect the absorption and utilization of exogenous sterols by *P. sojae*.

### Other *PsSCPs* Transcript Levels in *PsPTL* Mutants

Because the other three PsSCPs also have conserved SSD and Patched domains, it is plausible that they may have functional redundancy with PsPTL (PsSCP3). The transcription levels of *PsSCPs* genes in *PsPTL* mutants and the wild-type strain were then determined. The transcript levels of *PsSCP1* increased insignificantly or even decreased slightly in transformants, compared to the EV strain. Regarding *PsSCP2* transcription, although ΔP-11 and ΔP-23 showed a slight increase (relative expression fold change <4) compared to the wild-type P6497 and EV transformant, ΔP-8 and ΔP-14 were not significantly different from the wild-type. Furthermore, transcript levels of *PsSCP4* in ΔP-11 and ΔP-14 were slightly increased with the relative expression fold change being less than three times, whereas ΔP-8 and ΔP-23 showed no significant difference compared to the wild-type or EV (Figure 4).

## Discussion

Hedgehog signaling pathway plays important regulatory roles in the various vital developmental process in many vertebrates and invertebrates, which had shown that the abnormal activation of Hh signaling could cause human developmental defects and major diseases, such as cancer (Pospisilik et al., 2010; Skoda et al., 2018; Jiang, 2021). However, Hh-related genes are absent in oomycetes, presumably due to gene loss, which means that the Hh pathway is incomplete in oomycetes (Bürglin, 2008). Ptc protein is a receptor of Hh protein, which belongs to SCPs, a family having key roles in different aspects of lipid transport, homeostasis, metabolism, and sterol-linked signaling pathway, which involves in the growth and development of vertebrates and *Drosophila* (Kuwabara and Labouesse, 2002; Cohen, 2003; Jackson et al., 2020). Four putative proteins containing SSD and Patched domains were found, which means that there may be Ptc or Ptr homologs in *Phytophthora*. It indicates that the most of members in Hh signaling pathway were lost during evolution in oomycetes, while Ptc or Ptr homologs may be essential for oomycetes, so they have been retained in the long evolutionary process. To date, the biological functions of SCPs in oomycetes have not been reported.

We found that the conserved N-terminal domain of NPC1 protein (NPC1-N) exists in SCP1, 2, and 4, but not in SCP3. This means that SCP1, 2, and 4 belong to NPC1 homologs, while SCP3 may be the PTL protein in *P. sojae* and *P. capsici*. Therefore, we speculate that the four SCPs may have some functional differentiation in *Phytophthora*, and the function of PTL may be distinct from the other three SCPs function in some pathways. The expression level of *PsPTL* gene was higher in the hyphae stage, indicating that it may play a greater role in the hyphae stage. Moreover, we used GFP tagging at the C terminus of PsPTL protein (PsPTL-GFP) to visualize the localization of PsPTL in *P. sojae* hyphae. We found that it mainly distributed in the cytoplasm with a punctate pattern, which indicates that it may express in some endosomal structures in the cytoplasm, and may participate in the transport of certain substances, like the Ptc homologs in other species. Sometimes, the same tag may induce different subcellular localizations depending on whether it appears on the C terminus (C') or amino terminus (N') of protein (Weill et al., 2019). However, we did not obtain transformants that could express relatively strong fluorescence when GFP was fused into the N' of PsPTL (GFP-PsPTL). We speculate that the overexpression of PsPTL may be toxic to the hyphae growth if GFP-PsPTL is functional, while PsPTL-GFP may not be the functional form. Furthermore, fusion proteins are different from their native form and may suffer from impaired activity, reduced stability, wrong targeting, unnatural topology, and so on (Terpe, 2003; Dave et al., 2016). Hence, the endogenous PsPTL locus should be marked to better reflect the location of protein, which needs to be further studied in the future.

The biological characteristics of *PsPTL* mutants were determined in order to explore the function of *PsPTL* in different growth and development stages of *P. sojae*. In this study, the transformant in which the *PsPTL* deletion was not successful was included as a negative control (EV). Because the CRISPR/Cas9 treatment or the transformation itself might cause some off-target deleterious mutations (Schaefer et al., 2017), a comparison of traits between mutants and EV transformant is also particularly important. Additionally, the *PsPTL* mutants were slightly different from each other in phenotypes on Plich medium, which may be caused by off-target impact during the protoplast transformation process, rather than the deletion of the *PTL* gene itself (Miao et al., 2018). However, in terms of overall trends, the *PsPTL* deletion was found to have no significant effect on the development and virulence of *P. sojae* under the conditions assayed. It indicates that *PsPTL* may not be an indispensable gene in the growth or pathogenic process of *Phytophthora*.

In addition, it appears that *PsPTL* is also not essential for *P. sojae* to cope with external stresses such as temperature, pH, oxidative and osmotic pressure, and to respond to exogenous sterol treatment under the conditions assayed. We wondered whether the other three *SCPs* genes may have functional redundancy with *PTL*, and the transcription levels of *PsSCPs* genes in *PsPTL* mutants were determined. Although, the other three *PsSCPs* showed some differences in expression level among *PsPTL* mutants, and the change level was not particularly prominent, which does not completely rule out the possibility that these genes do not have functional complementarity. Therefore, the specific functions and involving pathways of these proteins need more in-depth research.

Although under the test conditions of this experiment, *PsPTL* may not have a prominent contribution to the growth and stress sensing of *P. sojae*, it cannot be ruled out the possibility that *PsPTL* plays a special role under a certain condition. Thus, a more complete understanding of the respective functions of *PsPTL* requires further investigation. In addition, although *Phytophthora* is a sterol-deficient organism (Madoui et al., 2009; Jiang et al., 2013), exogenous sterols can significantly promote the growth of hyphae and the production of spores (Langcake, 1974; Nes, 1987; Galanina and Konova, 2000; Stong et al., 2013). We should also pay attention to the other proteins that are presumed to have SSD, and further explore their biological functions to analyze the mechanism of uptake and utilization of exogenous sterols in *Phytophthora*.

## Conclusion

This study found that four putative proteins containing SSD and Patched domains exist in *Phytophthora*, one of which belongs to PTL protein, while the other three proteins belong to NPC1 proteins containing the N-terminal NPC1 domain. We knocked out the *PsPTL* gene to explore its biological function, and found that the *PsPTL* gene has no significant effect on the mycelial growth, spore production, and pathogenicity of *P. sojae*. Further investigations showed that *PsPTL* is also not essential for *P. sojae* to cope with external stresses such as temperature, pH, oxidative and osmotic pressure, as well as response to exogenous sterols. The transcript levels of the other three *PsSCPs* did not significantly change in *PsPTL* mutants, which indicates that there may be no functional redundancy between *PsPTL* and the other three genes. Our studies demonstrated that *PsPTL* is not an essential protein for *P. sojae* under the tested conditions.

## Data Availability Statement

The datasets presented in this study can be found in online repositories. The names of the repository/repositories and accession number(s) can be found in the article/Supplementary Material.

## Author Contributions

XL and ZX conceived and designed the experiments. ZX, WW, and JS performed the experiments. JZ and XZ contributed reagents, materials, and analysis tools. XL supervised the work. ZX wrote the main manuscript. XL and WW revised the manuscript. All authors contributed to the article and approved the submitted version.

## Funding

This work was funded by the National Natural Science Foundation of China (No. 31972304) and the Innovation Capability Support Plan of Shaanxi Province (2020TD-035).

## Conflict of Interest

The authors declare that the research was conducted in the absence of any commercial or financial relationships that could be construed as a potential conflict of interest.

## Publisher’s Note

All claims expressed in this article are solely those of the authors and do not necessarily represent those of their affiliated organizations, or those of the publisher, the editors and the reviewers. Any product that may be evaluated in this article, or claim that may be made by its manufacturer, is not guaranteed or endorsed by the publisher.

## Footnotes

^1^ http://genome.jgi.doe.gov/

^2^ http://fungidb.org/fungidb/

^3^ https://www.ncbi.nlm.nih.gov/

^4^ https://indra.mullins.microbiol.washington.edu/sms2/orf_find.html

^5^ http://www.cbs.dtu.dk/services/TMHMM/

^6^ www.megasoftware.net

^7^ http://smart.embl-heidelberg.de/

^8^ https://mycocosm.jgi.doe.gov/pages/search-for-genes.jsf?organism=Physo3

^9^ http://genome.jgi.doe.gov/Phyca11/Phyca11.home.html
