# Supplementary material for: A Patched-Like Protein PsPTL Is Not Essential for the Growth and Response to Various Stresses in Phytophthora sojae
Source: Front Microbiol. 2021 Oct 7;12:673784. doi: 10.3389/fmicb.2021.673784 (PMC8530017; doi:10.3389/fmicb.2021.673784)
Supplement: Supplementary file 1 [file Data_Sheet_1.docx]

Supplementary Material

**Supplementary Table S1. The information of SSD-containing protein sequences in different species used for phylogenic analysis**

| Protein name | Accession number in JGI | Accession number in FungiDB | Accession number in NCBI |
| --- | --- | --- | --- |
| *Phytophthora capsici* SCP1 | Protein ID: 543794 | PHYCA_543794 | — |
| *Phytophthora capsici* SCP2 | Protein ID: 54680 | PHYCA_54680 | — |
| *Phytophthora capsici* SCP3 | Protein ID: 563749 | PHYCA_563749 | — |
| *Phytophthora capsici* SCP4 | Protein ID: 506722 | PHYCA_506722 | — |
| *Phytophthora sojae* SCP1 | Protein ID: 466676 | PHYSODRAFT_466676 | — |
| *Phytophthora sojae* SCP2 | Protein ID: 318600 &  Protein ID: 549457 | PHYSODRAFT_318600& PHYSODRAFT_549457 | — |
| *Phytophthora sojae* SCP3 | Protein ID: 322952 | PHYSODRAFT_322952 | — |
| *Phytophthora sojae* SCP4 | Protein ID: 389466 &  Protein ID: 564545 | PHYSODRAFT_389466& PHYSODRAFT_564545 | — |
| *Phytophthora ramorum* SCP1 | — | PSURA_83385 | — |
| *Phytophthora infestans* SCP1 | — | PITG_10901 | — |
| *Peronospora effusa* SCP1 | — | DD237_004290 | — |
| *Phytophthora parasitica* SCP1 | — | PPTG_17607 | — |
| *Phytopythium vexans* SCP1 | — | PVE_G008939 | — |
| *Phytophthora ramorum* SCP2 | — | PSURA_85726 | — |
| *Phytophthora infestans* SCP2 | — | PITG_21540 | — |
| *Peronospora effusa* SCP2 | — | DD237_005964 | — |
| *Phytophthora parasitica* SCP2 | — | PPTG_07417 | — |
| *Phytopythium vexans* SCP2 | — | PVE_G010355 | — |
| *Phytophthora ramorum* SCP3 | — | PSURA_83375 | — |
| *Phytophthora infestans* SCP3 | — | PITG_10888 | — |
| *Peronospora effusa* SCP3 | — | DD237_004274 | — |
| *Phytophthora parasitica* SCP3 | — | PPTG_17593 | — |
| *Phytopythium vexans* SCP3 | — | PVE_G003472 | — |
| *Phytophthora parasitica* SCP4 | — | PPTG_07422 | — |
| *Homo sapiens* PTCH1 | — | — | AAC50550.1 |
| *Homo sapiens* PTCH2 | — | — | AAD25953.1 |
| *Drosophila melanogaster* Ptc | — | — | NP_523661.2 |
| *Caenorhabditis elegans* PTC-1 | — | — | NP_495662.2 |
| *Caenorhabditis elegans* PTC-3 | — | — | NP_001122650.1 |
| *Homo sapiens* NPC1 | — | — | ANN44507.1 |
| *Drosophila melanogaster* NPC1 | — | — | CAB56505.1 |
| *Mus musculus* NPC1 | — | — | AAB63372.1 |
| *Caenorhabditis elegans* NPC1 homolog 1 | — | — | NP_508771.1 |
| *Caenorhabditis elegans* NPC1 homolog 2 | — | — | NP_498813.1 |
| *Saccharomyces cerevisiae* Ncr1p | — | — | ONH72921.1 |
| *Homo sapiens* PTR | — | — | NP_001030014.2 |
| *Drosophila melanogaster* Ptr | — | — | NP_001246145.1 |
| *Caenorhabditis elegans* PTR-6 | — | — | NP_494986.2 |
| *Drosophila melanogaster* Disp | — | — | AAF23397.1 |
| *Homo sapiens* DISP homolog 1 | — | — | NP_001356523.1 |
| *Homo sapiens* DISP homolog 2 | — | — | NP_277045.1 |
| *Homo sapiens* DISP homolog 3 | — | — | NP_065831.1 |
| *Caenorhabditis elegans* PTD-2 | — | — | NP_505396.2 |
| *Caenorhabditis elegans* CHE-14 | — | — | NP_491673.1 |
| *Homo sapiens* DHCR7 | — | — | NP_001157289.1 |
| *Mus musculus* DHCR7 | — | — | CAJ18485.1 |
| *Rattus norvegicus* DHCR7 | — | — | NP_071784.1 |
| *Homo sapiens* HMGCR | — | — | NP_001124468.1 |
| *Drosophila melanogaster* HMGCR | — | — | NP_732900.1 |
| *Mus musculus* HMGCR | — | — | AAH85083.1 |
| *Homo sapiens* SCAP | — | — | AAH20987.1 |
| *Drosophila melanogaster* SCAP | — | — | NP_788277.1 |

**Supplementary Table S2. Primers and sgRNA sequences used in the study**

| Primer | Sequence (5’-3’) | Application |
| --- | --- | --- |
| P1-F | CCAACGACCACACACGATAC | Amplify *PsSCP3* transcripts for qRT-PCR quantification |
| P1-R | CGACAATGCCCAGGTACATG |  |
| P2-F | CAGGATGGTGCTAAGAACGC | Amplify *PsSCP1* transcripts for qRT-PCR quantification |
| P2-R | TCCAGCCTTGAAGACGATGT |  |
| P3-F | TTTGCGTTCGGTTCCATCTC | Amplify *PsSCP2* transcripts for qRT-PCR quantification |
| P3-R | AATGATGTCGTACTTGCCGC |  |
| P4-F | GCTTTGCACCGAGTTGAAGA | Amplify *PsSCP4* transcripts for qRT-PCR quantification |
| P4-R | GGAAACAAACGCCACAGAGT |  |
| P5-F | TCAAGTCGGACGCCTTCTA | Amplify *PsRPL13α* transcripts to standardize qRT-PCR quantification |
| P5-R | AGCAAGTGACTCAATGGGAAT |  |
| P6-F | CTCCAAGGGCTCGTCCAA | Amplify *PsRPS* transcripts to standardize qRT-PCR quantification |
| P6-R | GCCAGCATCCCTCCAAAG |  |
| P7-F | ATCGATAAGCTTGATGCCCAGTCCCGTCTTCTT | Amplification of the 1000 bp upstream of *PsPTL* |
| P7-R | ATCTTGTTCAATCATGGCTCGGAGAGGGAGGGA |  |
| P8-F | ATGATTGAACAAGATGGATTGCACGCAGGTTCTCC | Amplification of *NPTII* |
| P8-R | GACACCTAGCACCACTCAGAAGAACTCGTCAAGAA |  |
| P9-F | GTGGTGCTAGGTGTCAACAATACTTCTCTGGCCC | Amplification of the 1000 bp downstream of *PsPTL* |
| P9-R | CTGCAGGAATTCGATGAGATTGACGAAGGTAACC |  |
| P10-F | ACGTCTTGGGGTGAGAGATG | Amplification beyond the 1000 bp upstream of *PsPTL* and partial *NPTII* to verify homozygous knockout transformmants |
| P10-R | CTCGCTCGATGCGATGTTTC |  |
| P11-F | GGACCGCTATCAGGACAT | Amplification beyond the 1000 bp downstream of *PsPTL* and partial *NPTII* to verify homozygous knockout transformmants |
| P11-R | TGTAGGTGGCACGAGCAATT |  |
| P12-F | ATGGACCAGAATGGCGTCCC | Amplification of the partial sequence of *PsPTL* |
| P12-R | CGGACAATCGCACTGACCTT |  |
| P13-F | GATAGGCCTCCGCGGACTAGTATGGCGCGCGCCTTCTAT | Amplification of the full coding sequence of *PsPTL* without stop codons |
| P13-R | CCTTGCCCATCTTGAACGCTCTCACTTCGTCCT |  |
| P14-F | AGCGTTCAAGATGGGCAAGGGCGAGGAACTGTTCACTG | Amplification of the sequence of *eGFP* |
| P14-R | AGAAGTAGGCACCGGTACCGGGCCCTCAACGCGTTCCGGAGTT |  |
| sg2425-re | TCACGAACATGGACTGGTTG | sgRNA sequence |
| sg2230-re | ACGGGTAGAACAGCGCATTG |  |
| sg2232-re | CAACGGGTAGAACAGCGCAT |  |

**Supplementary Table S3. The putative PsSCPs predicted by Hidden Markov Model in *P. sojae* genome of JGI database**

| No. | Sequence Description | E-value | score | Location in *P. sojae* genome |
| --- | --- | --- | --- | --- |
| 1 | jgi\|Physo3\|391581\|gw1.1.4527.1 | 5.00E-99 | 336.4 | Scaffold 1: 9466598-9471508(-) |
| 2 | jgi\|Physo3\|455764\|gw1.1.14454.1 | 5.00E-99 | 336.4 | Scaffold 1: 9466598-9471508(-) |
| 3 | jgi\|Physo3\|466676\|e_gw1.1.14454.1 | 5.00E-99 | 336.3 | Scaffold 1: 9466592-9471508(-) |
| 4 | jgi\|Physo3\|469181\|e_gw1.1.4527.1 | 5.00E-99 | 336.3 | Scaffold 1: 9466592-9471508(-) |
| 5 | jgi\|Physo3\|431687\|gw1.1.10666.1 | 5.20E-99 | 336.3 | Scaffold 1: 9466595-9471508(-) |
| 6 | jgi\|Physo3\|294608\|fgenesh1_pg.1_#_1781 | 5.20E-99 | 336.3 | Scaffold 1: 9466592-9471508(-) |
| 7 | jgi\|Physo3\|309803\|fgenesh1_pm.1_#_1508 | 5.20E-99 | 336.3 | Scaffold 1: 9466592-9471508(-) |
| 8 | jgi\|Physo3\|467627\|e_gw1.1.10666.1 | 5.20E-99 | 336.3 | Scaffold 1: 9466592-9471508(-) |
| 9 | jgi\|Physo3\|322964\|gm1.2539_g | 7.80E-99 | 335.7 | Scaffold 1: 9466592-9472012(-) |
| 10 | jgi\|Physo3\|420679\|gw1.9.2308.1 | 6.70E-82 | 279.6 | Scaffold 9: 3042704-3044944(-) |
| 11 | jgi\|Physo3\|429130\|gw1.9.2742.1 | 9.10E-32 | 113.5 | Scaffold 9: 3043418-3045400(-) |
| 12 | jgi\|Physo3\|318600\|fgenesh1_pm.9_#_457 | 9.10E-32 | 113.5 | Scaffold 9: 3043245-3045400(-) |
| 13 | jgi\|Physo3\|305520\|fgenesh1_pg.9_#_594 | 1.20E-171 | 576.9 | Scaffold 9: 3040964-3045400(-) |
| 14 | jgi\|Physo3\|338979\|gm1.18554_g | 1.20E-171 | 576.9 | Scaffold 9: 3040964-3045400(-) |
| 15 | jgi\|Physo3\|347884\|estExt_Genemark1.C_9_t20314 | 1.20E-171 | 576.9 | Scaffold 9: 3040924-3045400(-) |
| 16 | jgi\|Physo3\|361948\|estExt_fgenesh1_pg.C_9_t20094 | 1.20E-171 | 576.9 | Scaffold 9: 3040924-3045400(-) |
| 17 | jgi\|Physo3\|318599\|fgenesh1_pm.9_#_456 | 2.90E-169 | 569 | Scaffold 9: 3040964-3044944(-) |
| 18 | jgi\|Physo3\|352652\|estExt_fgenesh1_pm.C_90449 | 2.90E-169 | 569 | Scaffold 9: 3040924-3044944(-) |
| 19 | jgi\|Physo3\|523358\|e_gw1.9.2561.1 | 2.90E-169 | 569 | Scaffold 9: 3040964-3044944(-) |
| 20 | jgi\|Physo3\|549458\|estExt_Genewise1.C_9_t60115 | 2.90E-169 | 569 | Scaffold 9: 3040924-3044944(-) |
| 21 | jgi\|Physo3\|564542\|estExt_Genewise1Plus.C_9_t50491 | 2.90E-169 | 569 | Scaffold 9: 3040924-3044944(-) |
| 22 | jgi\|Physo3\|425964\|gw1.9.2561.1 | 2.90E-169 | 569 | Scaffold 9: 3040967-3044944(-) |
| 23 | jgi\|Physo3\|426884\|gw1.9.2616.1 | 4.60E-117 | 396.1 | Scaffold 9: 3040967-3043171(-) |
| 24 | jgi\|Physo3\|521438\|e_gw1.9.2616.1 | 4.60E-117 | 396.1 | Scaffold 9: 3040964-3043171(-) |
| 25 | jgi\|Physo3\|549457\|estExt_Genewise1.C_9_t60114 | 4.60E-117 | 396.1 | Scaffold 9: 3040924-3043171(-) |
| 26 | jgi\|Physo3\|564541\|estExt_Genewise1Plus.C_9_t50490 | 4.60E-117 | 396.1 | Scaffold 9: 3040924-3043171(-) |
| 27 | jgi\|Physo3\|521992\|e_gw1.9.2872.1 | 7.20E-77 | 262.9 | Scaffold 9: 3040964-3042652(-) |
| 28 | jgi\|Physo3\|564540\|estExt_Genewise1Plus.C_9_t50489 | 7.20E-77 | 262.9 | Scaffold 9: 3040924-3042652(-) |
| 29 | jgi\|Physo3\|431511\|gw1.9.2872.1 | 3.00E-76 | 260.9 | Scaffold 9: 3040967-3042415(-) |
| 30 | jgi\|Physo3\|549456\|estExt_Genewise1.C_9_t60113 | 5.30E-70 | 240.2 | Scaffold 9: 3040924-3042415(-) |
| 31 | jgi\|Physo3\|309793\|fgenesh1_pm.1_#_1498 | 1.70E-132 | 447.2 | Scaffold 1: 9429809-9432946(-) |
| 32 | jgi\|Physo3\|322952\|gm1.2527_g | 1.70E-132 | 447.2 | Scaffold 1: 9429809-9432946(-) |
| 33 | jgi\|Physo3\|475300\|e_gw1.1.10520.1 | 1.70E-132 | 447.2 | Scaffold 1: 9429809-9432946(-) |
| 34 | jgi\|Physo3\|430525\|gw1.1.10520.1 | 6.10E-132 | 445.4 | Scaffold 1: 9429812-9432940(-) |
| 35 | jgi\|Physo3\|473433\|e_gw1.1.3229.1 | 7.70E-131 | 441.7 | Scaffold 1: 9429809-9432946(-) |
| 36 | jgi\|Physo3\|474409\|e_gw1.1.13793.1 | 7.70E-131 | 441.7 | Scaffold 1: 9429809-9432946(-) |
| 37 | jgi\|Physo3\|383674\|gw1.1.3229.1 | 1.70E-130 | 440.6 | Scaffold 1: 9429812-9432940(-) |
| 38 | jgi\|Physo3\|451330\|gw1.1.13793.1 | 1.70E-130 | 440.6 | Scaffold 1: 9429812-9432940(-) |
| 39 | jgi\|Physo3\|294598\|fgenesh1_pg.1_#_1771 | 2.90E-128 | 433.2 | Scaffold 1: 9429809-9432892(-) |
| 40 | jgi\|Physo3\|338983\|gm1.18558_g | 3.00E-173 | 582.2 | Scaffold 9: 3057011-3061471(+) |
| 41 | jgi\|Physo3\|347885\|estExt_Genemark1.C_9_t20318 | 3.00E-173 | 582.2 | Scaffold 9: 3057011-3061690(+) |
| 42 | jgi\|Physo3\|425529\|gw1.9.2536.1 | 2.30E-171 | 576 | Scaffold 9: 3057485-3061468(+) |
| 43 | jgi\|Physo3\|523692\|e_gw1.9.2536.1 | 2.30E-171 | 576 | Scaffold 9: 3057485-3061471(+) |
| 44 | jgi\|Physo3\|549459\|estExt_Genewise1.C_9_t60129 | 2.30E-171 | 576 | Scaffold 9: 3057485-3061690(+) |
| 45 | jgi\|Physo3\|564543\|estExt_Genewise1Plus.C_9_t60001 | 2.30E-171 | 576 | Scaffold 9: 3057485-3061690(+) |
| 46 | jgi\|Physo3\|305524\|fgenesh1_pg.9_#_598 | 3.70E-157 | 528.9 | Scaffold 9: 3057011-3061471(+) |
| 47 | jgi\|Physo3\|361949\|estExt_fgenesh1_pg.C_9_t20098 | 3.70E-157 | 528.9 | Scaffold 9: 3057011-3061690(+) |
| 48 | jgi\|Physo3\|389466\|gw1.9.969.1 | 3.80E-84 | 287 | Scaffold 9: 3057485-3059731(+) |
| 49 | jgi\|Physo3\|426591\|gw1.9.2598.1 | 2.60E-33 | 118.7 | Scaffold 9: 3057011-3059017(+) |
| 50 | jgi\|Physo3\|431618\|gw1.9.2879.1 | 1.50E-116 | 394.4 | Scaffold 9: 3059264-3061468(+) |
| 51 | jgi\|Physo3\|318604\|fgenesh1_pm.9_#_461 | 1.50E-116 | 394.4 | Scaffold 9: 3059264-3061471(+) |
| 52 | jgi\|Physo3\|352653\|estExt_fgenesh1_pm.C_90454 | 1.50E-116 | 394.4 | Scaffold 9: 3059264-3061690(+) |
| 53 | jgi\|Physo3\|522220\|e_gw1.9.2879.1 | 1.50E-116 | 394.4 | Scaffold 9: 3059264-3061471(+) |
| 54 | jgi\|Physo3\|549460\|estExt_Genewise1.C_9_t60130 | 1.50E-116 | 394.4 | Scaffold 9: 3059264-3061490(+) |
| 55 | jgi\|Physo3\|564544\|estExt_Genewise1Plus.C_9_t60002 | 1.50E-116 | 394.4 | Scaffold 9: 3059264-3061490(+) |
| 56 | jgi\|Physo3\|429278\|gw1.9.2753.1 | 3.00E-54 | 188 | Scaffold 9: 3060347-3061468(+) |
| 57 | jgi\|Physo3\|523581\|e_gw1.9.2753.1 | 3.00E-54 | 188 | Scaffold 9: 3060347-3061471(+) |
| 58 | jgi\|Physo3\|549461\|estExt_Genewise1.C_9_t60131 | 3.00E-54 | 188 | Scaffold 9: 3060347-3061690(+) |
| 59 | jgi\|Physo3\|564545\|estExt_Genewise1Plus.C_9_t60003 | 3.00E-54 | 188 | Scaffold 9: 3060347-3061690(+) |

**
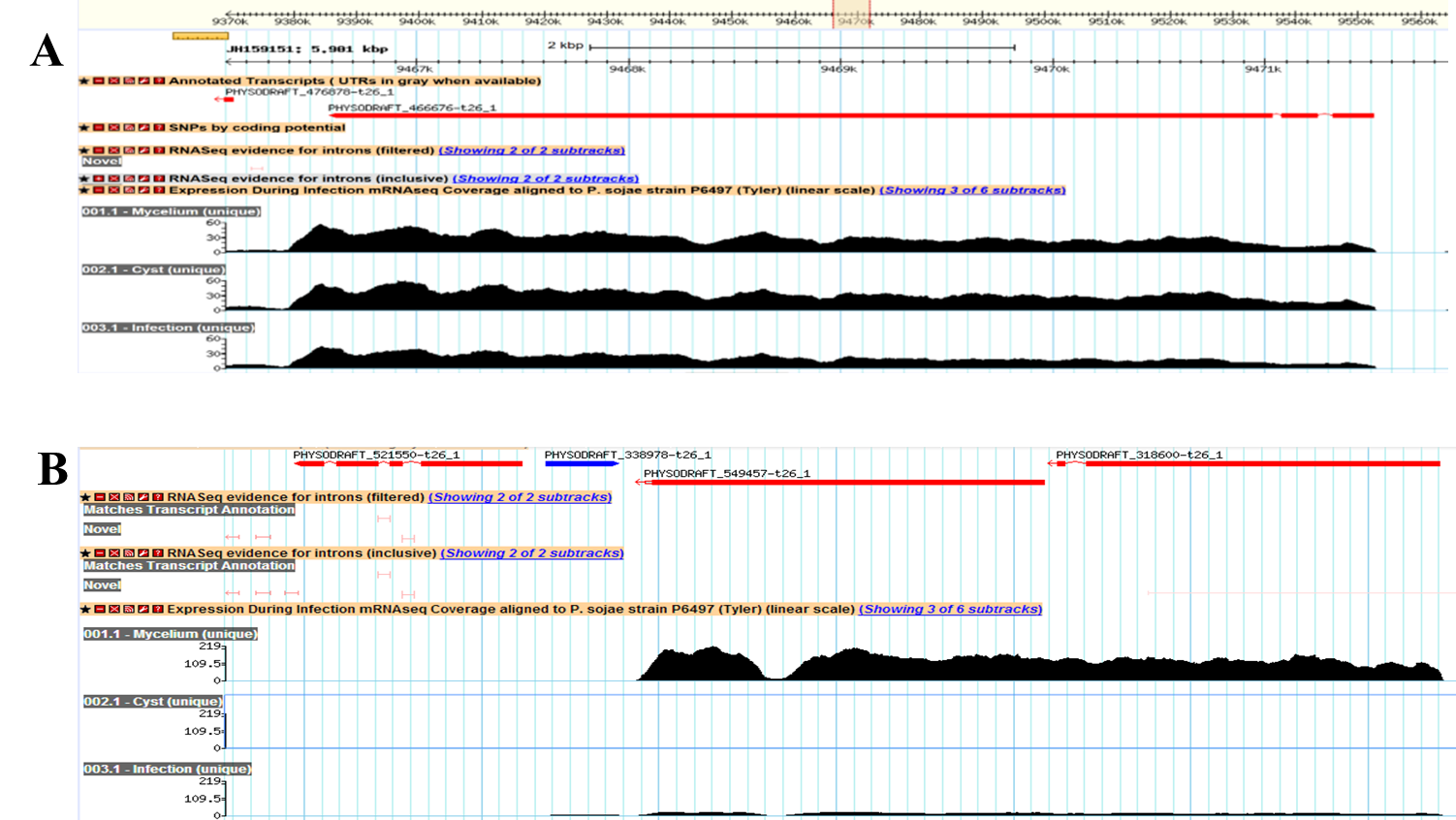
**

**
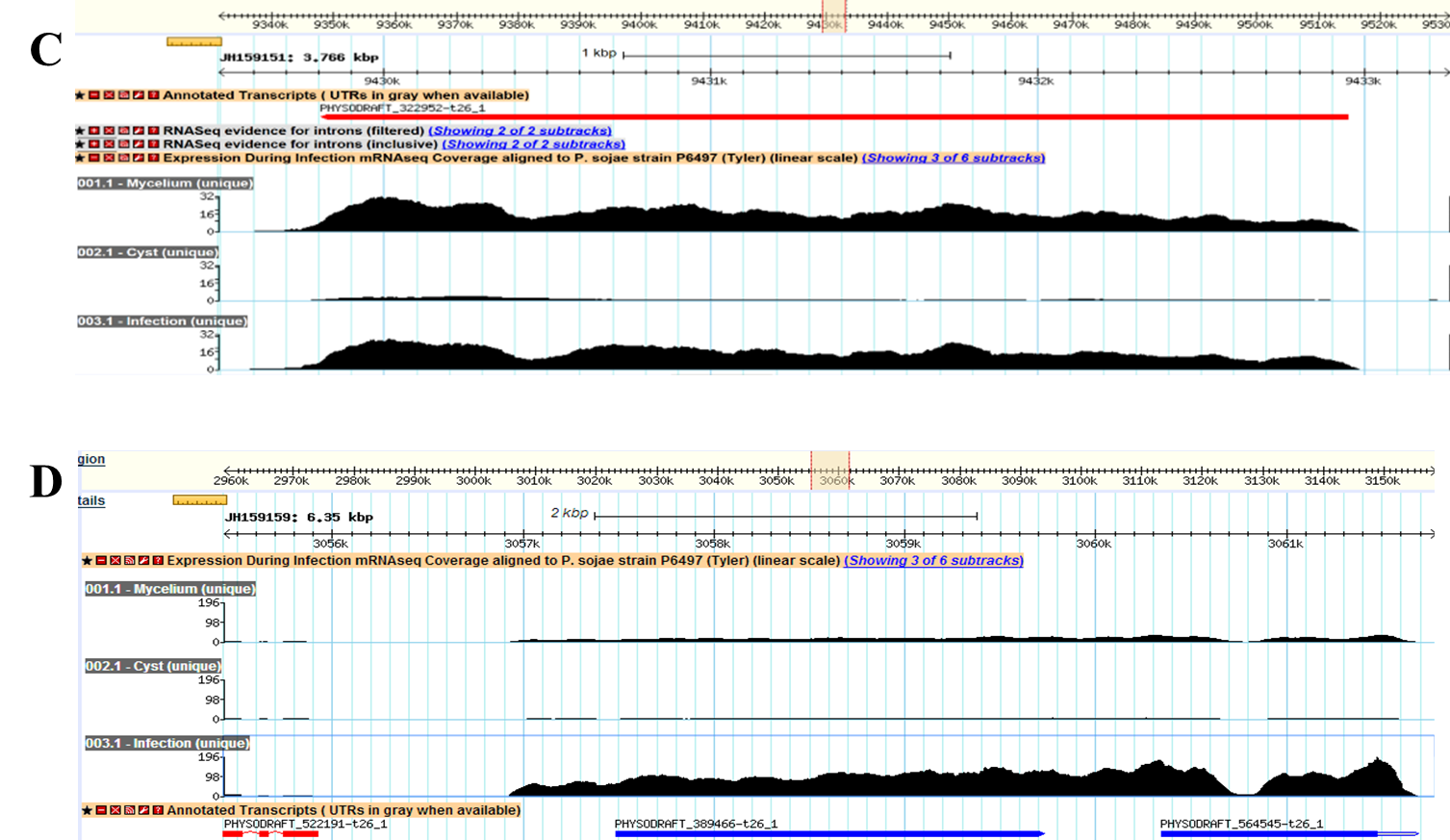
**

**Supplementary Figure S1. RNA-seq data of putative *PsSCPs* in *P. sojae* in FungiDB database. (A) *PsSCP1*, (B) *PsSCP2*, (C) *PsSCP3*, and (D) *PsSCP4*.**

**
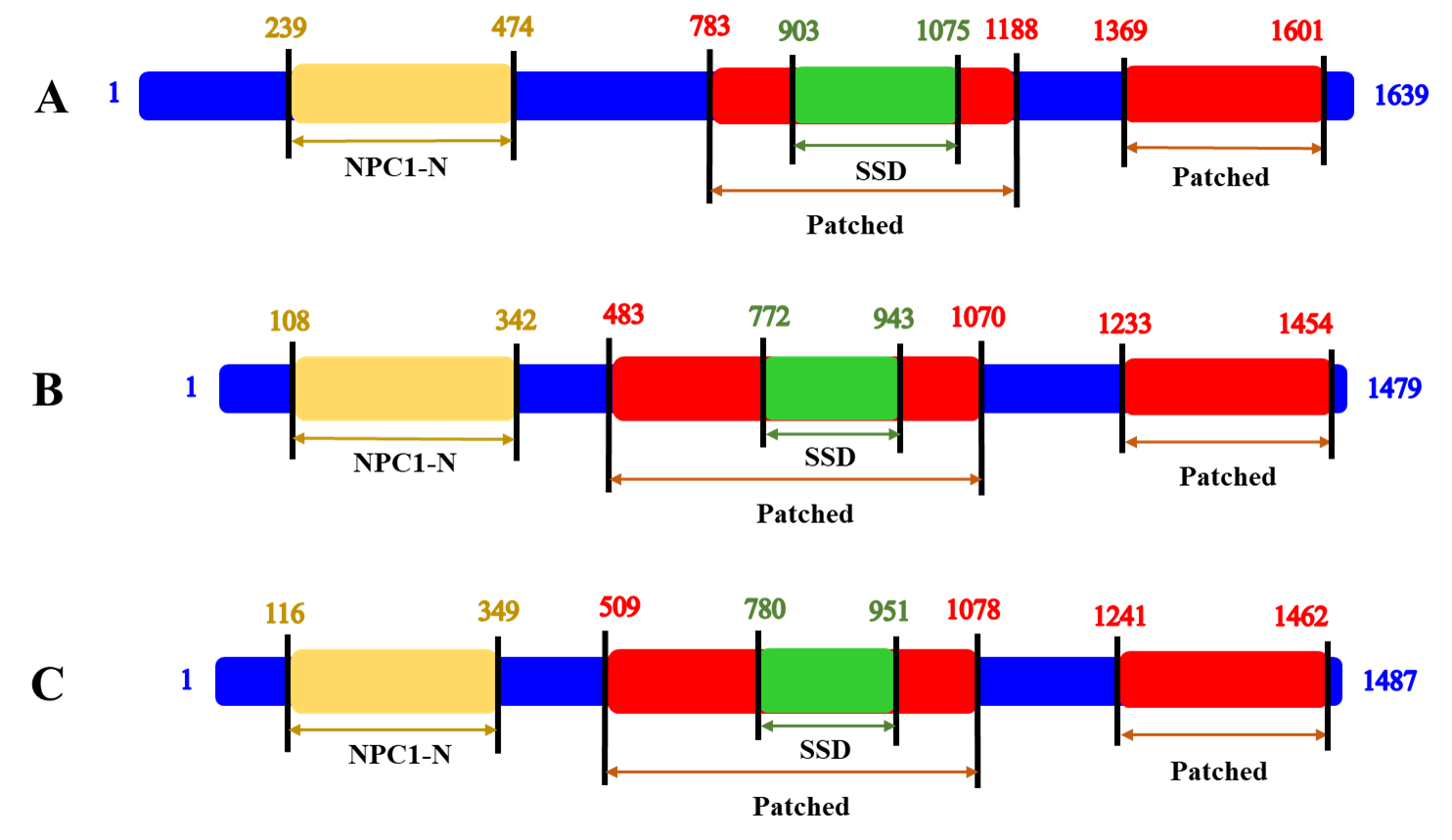
**

**Supplementary Figure S2. Sequence characteristics and conserved domains of putative PsSCPs.** **(A) PsSCP1, (B) PsSCP2, (C) PsSCP4.**

**Supplementary Figure S3. Phylogeny analysis of SCP orthologs in oomycetes with Patched proteins of other species.**


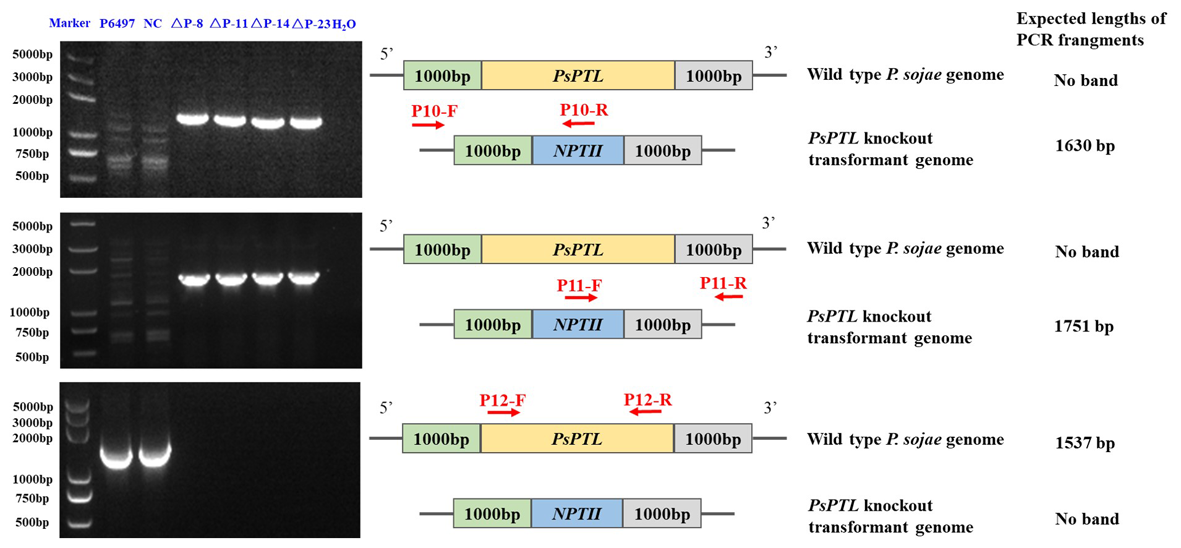


**Supplementary Figure S4. PCR verification of *PsPTL* knockout transformants.** Primer locations in the wild type or *PsPTL* mutant genomes are marked using red arrows. 5’ 1000 bp and 3’ 1000 bp indicate 1000 bp upstream and 1000 bp downstream regions of *PsPTL* gene (protein ID: 322952). The lengths of the *NPTII* and *PsPTL* genes shown are 795 bp and 3138 bp, respectively. The expected lengths of each PCR fragment are marked on the right. Lanes from left to right means the templates: Marker, wild-type P6497, negative control (NC), mutant △P-8, mutant △P-11, mutant △P-14, mutant △P-23, and H_2_O.

**> Coding sequence (CDS) of *PsSCP1***

**ATGTGGCTGTTCGCGGCCATCAGCCTCGCGATCGCGACGCCGTGCTTCCGCGCGTGGCTCGAGGCGCTGTTCGCCGAGCTGTCCATCATCTTCTTCACGTACCTGCGGCTGGTGTCGGCCGGCCTGAGCCTCGTGCTGCTGCTGGCGCTGGCCTTCCACGGCCGCCGCTTCCTGCGCAGTCGTCGGGTCGCCAAGGCGCCGCGAGCGGGCGGCAGCGGCAGCCAAGCGGCGGATGAGGGATCCAAGGCGTCGCTGTCGGCCAAGACGGGCCTGGCGCGCGTCCACTGGCGGCGCGGCACCAGGCGCCGCGCGCTCATGCTGCGCGTCAAGAGCACGACCAGCGAGATCGCGGGCAGCGGCTTCTACCCGCCGCAGCTCTGGACCTCGTCGCTCTTCGCGCTGGCCATGCTGCCCATTATCAACAGCGTCGTGGTCGGGGGCGGAGGCGGCTCGAGTCTGCGCGGACTCAAGAGCGAGACGGCGGCCGCCGCCCAAACGGCTTTCGACGCCGACGCGGCGGCACTCGTGACGCTGCAGCAGCAGCTGACGACCTGCAAGTACTCGGGAGGCGACGACTGTCTTGACGACGTGACTGCCATCTCTGTGCTGGGGAGCTATCAGCAGGCGAGTGGATACTGCGTGGCCTTTGACGCGGCCTATGTGAATGTGACGACGGGGGCGGCACTACCGGCCCAGTACTTCCCCATCGGGGTGGAGGAGGCCCACGCCCAGGGCTTTGCCAACAACTTCTCGGCGTGGTCCGAGACCAACCAGGAGAAGTTCAAGACGGACTGCCCCCTGCTGTTCAACGAGACGGTGAGTGGAGACGGAGAGGGGCTGCTGTGCTGTACCGAGACGCAGTACGAGATGCTGAGCCTGCAGGTCCGGAAGCTGCCTGGAGAGTGCACGTCCTGCAAGCAGAACCTGCGCAACCTGTGGTGCCAGTTCACGTGCCATCCGAGCAACAGCCTGTTCGTGGACGTGACGCAGGTGCGGCTTATGGAGGGAGACGCGGACCACGCGGACGAGGTCTTCCCCGCCATCGAGGAGGCCACCTACTACGTGGGCAGTGACATGGTGCGCGACCTGCACGACTTTTGTGAGGCCGACTCGGGCTTCATGCCGCTGTTGTGCGGGATGAATGCCGATGGTAATTGCTCGACGACAGGCTCGGACATGTTGGGGTACCTTGGAGCGTACAGCTTTGACGGTGTGGGATCGCCCTCGCAGGTGATCTTCACTACGATGGAGCAGCTGTCAGCGGCTGAGCAGGAGGACAAGATCTGTGCCTGTGACAGCAGCAACACGACTGGCTGCTTTTCGCCGATGGATACGCGGCTTGAGTCGTGCGTGGATACGTGTGGCTCGCTCTGTGCTGTGAGTGATGATGACAGCCGGCAGTATCAGGCGGCTTGCTACAGTTCCGGCAGTACGTCGGCATCCGACGATTTGAGCACTGTCACTACAGCCACGACCAGCACCAGCGCTGCTGATAAGCTTGAGTCTTTACTGTCGGATTTGTCGTCCCGTGCGGAGGGTGGTAGTTTTGCCGTGCTAAACTATGTCCTTGCTGTTCTCGCATTCTTTGGCGCCACAGCGCTTGCGCTCGGCTTCGCGTACTCGACGCGGTACGGCAGGAAGAAGCGCCAGTCTGTGCTGGACGACCCTGTTAACGGTTTGGGATCTGGAATGCTGTCACTCGTGAATTTGGATCAGTTGAAGGGAATCGGACGTTGGGATGACCGGCTGACGATGCACTTGAAACGCTGGGGAGATTTCGTGGCCATGGGGAACCACCCGTTGTACATTATTCTACTGTCGTTGATGGTTGTCGTTTGCTGCTCGAGTGGGTTGATCCGCATGGAAGTGGAGACGGACTCCATGAAGCTGTGGGTTTCCGGTAGGAGCTCTGTTTTTCAGGAGAGGACGCGCTTCGGCGAGATGCTTGGGCCAGTGGATCGTATGGAACGACTGGTCCTTGTCACTAAGGACGGCGGCGCAGTGACCAGACCAGCGTATATCAAGGAGGCGATCCGCTTGCAGCAAGTCATTGGAAGCGAGGTTGCCGCTGACAGTATTACACTGAGTGAGATCTGCGTCAAAGACGCCTCGAGCTCTCCATGCCAGGTGAATTCCGTAACGCAGTACTTCCAGAACAGCATGGATCACTTCAACATGTACGATGCGTATGGGCTCGTAGGCAAGCACTTGAGCAACTGCGCTAATGCTCCGGAACGAGCGGACGGTAATGTGTGCAGTGAGCTGCAAGTGCAACTCAATGCCTCGGGTGCTTCGCTTCCGACGAGTATGAGCGGCTGCCCTTGTGCGTCGTCGTTTGGGGTGCCGATGGCTGAGCTGGAGAAGTACCTGGGCGGATTGAGTACTGACGGTGGATCATTGAATGCCAGTGCTTATCTGGAGCAAGCGACAACTCTCTTTTCAACTGCCATGGTGACTAACCACCAGGATGGTGCTAAGAACGCAGATGCAATCGCTTGGGAACGCGCGTATATCGCGCGGATGGAGAAAGAATCGGATACCAACACGATGTATGATATCTATTATGCTGCAGAGGTGTCTGCTGACGACGAGTTCGTGGCTGCTTCCAACTTGGACATCGTCTTCAAGGCTGGAATTGCAGGCTTTCTCTTTATGTTTGTTTACGTGGTCATTGGACTGAACCACTGGAAGCTGGACTACCGCTTCTTCCATTCGTCAAAGATCGGTGTGGGTTTCATGGGCGTTGCGTGCATCCTGATGGCCGTGGGCGGGACCTTGGGTATCTTTGCGTGGACTGGAGTAAAGCTTCAGATCGTGACGCTCGTTGTGATGCCGGTGGTTGTACTTGCGATCGGCACTGGAAACATCTTCCTGATCCTGCATGCCGTCGACCTGAAGCAAGAAGAGTTGAAGATGGAGCAGCGCTCACTGTTTGTGGGTCTTGAAGACAACGATTTCGGTATTCATGAGATCACGTGTGTGCTGCTGTGCGAGGCAACGGGTTATATCGGCCCCAGCATGATCGCGACGTGCGTATGCGAGTGCTGTATCGTTGCTTTTGCAGCATATTCGACCATGCCAGCTGCACAGTGGCTCGCTGGATCGTTGGTGCTTGGGCTCGCAGCCAGTTTTGCTTTGCAAATGACCTTGTTCCTCGCCATCGTAGCGCTGGACAAGCGTCGTGAGCTCAGCGGCACGTACGATGTGATCTGCTGTAAGCGGGCATCGTTCGCTCGCCGCCCTCGCCTCTCCGAAGACGAGACGACTGCTGCGACCGAAAACTCGTCGTTCCCTGGAAGCACTATTTCACTGCCCGACTTGAACTTGATGAACCGATGCGTTGCTGGATACATCCATGTGCTGCTCAAGAAGGTGTCGAAGGTGTTGGTGCTCCTCGTGTTTGCCGCGTGTACTCTGGCGGCGATTGTCTCCATCGAGGCAATGGATCGCGGTCTTTCACCGAACTCGTTCATCCCGACGAACTCGTATCTGCACGCATACTACCGTGCAGTGGACGAAAATGACTTGTCGACGAAGGAGTTCCCTGCCTATTTCGTTGTTGAAGCTGGCTACGGAAGCAACCCTACCGGATTCAACGATCTCGCCAACGATGCGGAAGCTCAATGCAAACTGTGCTCGTCGAAGGAGTTCTGCGACGACTTGTCGATCCCGAATATTCTCAGTGCGTTGGTTGCCGCTGGGGAAAGCAACGTCACGTTTTTCAAGGACGGCACTGTTGTTGGATCATGGTTGGACGACTTCTGGAGCTTTGTTGACCCAGCCAGTGAGTGCTGCCGCGTCGATGCGGAGAACGACTACTCGTATTACGCTATTCTACCGGAAGAGAGCAGCGCTGAATATGTGCTCAAGCGCGCGTCCAACGCGCCATCGTGTCTCGCTGACTCAAGTGCGGTGCTCTCAGTGCCCGATGAATCGTTCATGTCGTTGTTCAGCATGTTCTCGACTGCGGCTGCTGGACCCTTGTGCTCGTACGCAGCAGGCACGCGCTACCATGGACAGCTCAGCGTTGATAGCCAACCTATTCCTGCCATGAGCAGTAGTGCTGCTGCGGGCGTGACTCTCAATGGCACTGGCTACGGCAGTGATGTGACGGCGTTTGTGTACAAGGTTCTGAGCACTACAGTCGGCTCGTCAAAGATTTCAGGAAGCCAAGAAGGAGCAATCGCAGCCTACTCGCAAGCTCAGCACATTGCCAAGTGGATCAGCGAGGAAACTGGCATCGATGTGTGGGCGTACTCCCCCGAGTACGTTTATCTGGATCAGTTCCACTCGGTTCGTCGTACTGCGTACATTGTGGTGGGTGTCGGACTCGCAGTGGTGTTCGTGCTGCAGAGTTTGGCTCTGGGGAGTTACTGGTATGGATTTGCGGTGACTTGTGTCGCTGCAGCCACGGTTGTCCAAGTCGCTGGCCTCATGATGCCGATGGGGGTCCCAATCAACTCGCTTTCGATTGTGAGCCTCTCGATCGCCGTCACCTTTTCCGTCGGATTCTCGGGGCATTTTGCTCGTCTTTTTGCCAAGGCTCGCACCATCACTGACGACTTGGGCTACTCACCTGGCGGCGATGCCTGCGTTCGGAAGGTGTTGGCGCAGCTTCTCGCGTCGTGGACGTTGGGCGTCGCCGTCTCCAAGTTCGTTGCCATCGCAGCGCTCGCGCTTGTCGCCACGCCCGTCTTCGAACCCGCTGGGAACTGTTTCTTCCGGACGCTGATGGCTGCAGCGGTGTGCGCGTGGCTGAACAGCGCCGTGCTGCTTCCCGTCGGGCTCAGCATTTGTGTGGATGCCACGGAAGGCCGCGTGCGTGACGTGAAGCCGACGAATGAAGAGGGCGGGGAGTACTCGCGTGAGAGCCCTTCGTCGTCATACCACACTGCGCCGCCGACTAGCAAGTACTGA**

**> Coding sequence (CDS) of *PsSCP2***

**ATGCGCGTCTCGGCCCTCGCGTCGCTGCTGCTGCCGCTGGGCGTGGCCCGCGCCGCCAGCTCCTCGAGCTCCACGACGTCGTCGGGCGTGTCGTCCGCCTCCAGCTACGTGCCGTGGAGCCTCGCCGACAACGCCACCAACCTGGCCATCATCGAGTCGCAGCTCACCATCTGCACGTACTCCAAGGTGGAGGAGTGCATCCAGGACCCCAAGCTCGTGCAGGAGCTCGGCTCGCTCTACCGCGCGCCGGGCTACTGCGTAGCCTTCGACTCGGCCTACGTCAACATCACCACGACCAGCGCTGCCATCCCCAACCGCTACTACCCGACGTCCGTGGAGGACGCGTACGACGCCGGCTTCTCCAACAAGTTCTCCGAGTGGTCGGACACCAACCGCGAGCAGTTCGAAGTGGACTGTCCGCTGCTCTACAATGAGACCATCGCCCTGGGCGACGACATGCTCTGCTGTACTGAGTCCCAGTACACAGGGCTGTCCACGCAGGTGCGCATGATCCCCGGTCTGTGCTCGGCCTGCAAGGAGAACCTGCGCAACATCTTCTGCCAGTTCACGTGCAACCCGAACAACAGCATGTTCCTGGACATCAACGAGGTGCGCATCATGTCGGGCGACGACGAGCACGAAGGTCAGATCTTCCCAGCGGTGGAGGAGGCAACGTACTACGTGGGCAAGGACTGGATCCGCGACATCTACGACTTCTGCGAAGACGACAGCTCCTTCTCGCTGCTGTGCAACCCCAACCAGGACTGTACCGACGGCTACGGCCTCATGGAGTACATGGGCAAGTACGCCTACAACAGCATCGGGTCCCCAGAGCAGATCAACGTGACAACTATGGATCAGCTTTCGGAGGAGCTGCAAATGACCGAGTTCTGCCATTGCGACTACGCCAACGAGACCAATTGCATTTCGCCCATGAACAACAAGATGACGTCGTGTGTGGGCGTCTGCGGCTCGCTTTGTGCCGTTAGCTCCGACGATACGCGCACGTACACGGATGCTTGCTACGGTGCCAAATCCGCGTCCTCTTCCGGTAGTGGCAGCGTCGTGGACAACGATGACGACGCGACTTGGATGGAGCTCAACGAGTACCTCGCCAACAACCTGCCCGCGACGGATTGGACCCCGTTCAACTACTTCCTCGTGATCTTTGGAGGCGTGGTGGGTGTGCTGCTGATTGTGGGCTTCATCGTCGCCATGCTCCGTGAGAGGAGGTACAGCGCAGTCGAGCGTAACTCGGGCACTCCGCAGGTAGTTGGCCCTAACACACCAGACTTCCATGGCGTGGCGAGCGCGATGGCTGGCAGCGTCGGATACTTGAGCTTCTTGGACGATCTGATGACAAACAAGCTGCGGTTGTGGGCGGCGTTCGTCTCCAAGGGTAACCGACCGAAGAAGATCACCCCGATGGTGCTGTGCGTTGTCGTCATTTGCGCCGTCGGCTTGTACAACATTGATATCGAAACGGACCCGATCAAGCTGTGGGTTTCTACGTCAAGTACTTCGTACGAGCAGCGCCAACATTATGGCGAGATCTTCAACCCGTTCTACCGCTCGGAGCAGATCATTATGGTCCCGAAGGACGGTGGCAACATCTACCGCTCGTCGATCATCAAGGAGGCCATCCGCGTGCAGACTGTGGCAGCCAACGTGACGTACACTTCGGACGACGGTGACGAGACGATTACTCTGGACGACATTTGCTGGAAAGCTACGGGCACGGGCTGCACCGTCAACTCGATCACGCAGTACTTCCAGAACAACATGGAGCACTTCGAGTTCTACGAGAAGTATGGATTGGAGCTGGAGCACTTTAGTAACTGCCTTTACTCTCCGACCACGTCGGACGTGGCGCTTTGCACCGAGTTGAAGAACGCTTTGGAGGATGGCGATTCGCTCCCGTCGAGCATGAGCGACTGCCCCTGCTTGTCGGCGTTTGGCTCGCCCATGAACCTGTACAACACGTACCTGGGTGGGTTCCCTGACGGCGCTGAGAGCAACTACACGCTGTTCCTCGAATCGGAAGCGTTTGTTTCTTCGTACCTCAACTACAACTACGCTGATGACGACAAGAACGAACCTGCCATCAAGTGGGAGCGCGAGTACATCAAGACGATGAAGAAGGAGGCTGCGTCTAACACGATCTTCGACGTCTATTTCTACGCCGAAATCTCCGTCAACGATGAGGTCGACGTTGAATCAAACAACGGAATGGGCCCTGTGGCGCTCAGTTACTGTTTGATGCTCATCTACATTTCGTTGGGCATTAATCGCATCAAGTTCAGTCGAGAGTTCTTCATTTCGTCCAAGATCCTGGCCGGATTCTGCGGTGTGATAAGTATTGCCTGCGGTGTGGCTTCGACGATCGGTATCTACATGTGGGCTGGAGTCAAGCTCCAGCTTATTATCATGGAAGTGGTTCCCTTCCTGTCGCTGGCCATCGGTGTCGACAACATCTTCTTGATCATCCACGCCATGACGGAGAAGGAAGATCAGCTCCGACGCGACCAGCCAAGTCTCTTTATTGGTCTGGAGCACAACCCCAAGGCGATCGAGGAGATCACAACGGTCATCTTGTCTGAGAGTATCGCGTACATCGGCCCCAGTATCTTCATGGCCTCCGCTGCTGAGTCGGTGGCATTTGCGTTCGGTTCCATCTCGGCGATGCCTGTCGTGCTGTGGTTCGCTGCGATGGCATGCTGTGCTGTGGCGATCAACTTTTGCTTCCAGATGACGTTCTTCCTCTCTGTTCTTACTCTGGACAAGCGTCGCGAGCTGAGCGGCAAGTACGACATCATTTTCAAGCGGGCGTCAGCGGTGGCAGCACAGGCACCCGCCGCTCCTGAGACTGTTCAGCATTCCTCAGAGCCGCTTGTGTCGCTGCAGCCCAAGACTCCAGCAGCGGACGATGTCCGTCCTTCTGTCACGCCCGAGAACAGCACGCTGACGGATGTCCTTGACTATTGCGTGGATGTCTACGCGTCGATATTAACCCACAAGCTTGTGAAGCTCGTCGTGCTGTTACTGTTCCTCGCGTGGACATTGTGGTCTATCTACTCGATGGAATCGCTGGACCAGGGCCTGCCGCAGAAGGAGGCCATGCCTTCAGACTCGTACATGATCGAGTACTTCAACGCACTGGATGTGTACCTCGCCACGGGTGTGCCGGTGTACTTCGTGGTCGAGACTGGCTATGGTCGGAACCCCGATGCTTGGTCACTGAACGATGAGAGTGTCGAGACGATCTTCTGCAAGTCCAAGGATATCTGCGGGACGTACTCAATTCCCAACATTATGAACGCATTGGCAAACGACGGTGACAAAACGAATACCCACATCAGCCCCGGCACGACCTACTCGTGGATGGATGACTTCTGGGGCTTCGTCAACCCTGACAGCGAGTGTTGCCGTGTGGATTCCGAGGGCGCCTACGTCCCGATCGAAACTGGCAACGACACGTACACGACTCTGCGCGCGGACGACGACACGTGCCTTGCTACGTCGGTGATGATTCCCCCCGTGCCTGAGGCCCAATACATGTCGCTCTTCAGTATGTTCGCGACGGCAAGTGCTGGTACGTCGTGCTCGTACGGTGGTGGTTCGATCTACCGTGGCCAGTTCAGTATCGACTCGGAGCCCATTCCGACCGTCAACGCCTCGACGCCTGCGGTGAAGATCAACAGCAGTGGCTACGGTGACGAGATCACGGCTTGGTCGTACATGGTGACGGGCACGTCGAACCCGACGCAGCAGCGCTACATCGACTCGTACAAGCAGAACCTTGCGGCGGCCGAGTGGATCAGCGAGAAGACTGGTGTGGACATCTGGGTGTACTCGCTGACGTACGTGTACTTCGAGCAGTACCTGACTGTGGTCGACGACGCCTACAAGCTCATCGGTCTGGCGCTGGCGGCTATCTTCGTGATCACGACGCTGTACCTGGGCAACGTGTTCTACTCGCTGGTGATCGCGTTGACGGCGACCAACATCGTCGTGCTCGTGCTGGGTCTGATGCAGCCGCTGGACATCATGCTCAACGGTCTGTCCATTGTGAACCTGATCATCGCGGCTGGCATCTCGGTTGAGTTCTGCGGTCACTACGTCCGGTTCTTCGCGAAGGCTCGTGGTACGGGCGACGAGCGAGCTCGCGATGCTCTTCGCCAGGTCTTGACGTCGGTTGTGTTTGGCATCACCATCACGAAGGTCATCGGTCTGAGTGTGCTGACACTCGCGGACTCGCGCGTGTTCAAGAAGTACTACTTCCGCATGTACATGATGGTCGTGGTGTGCGGTGTGCTCAACGGCATGTTGCTGCTGCCTGTGGTGCTGAGCACCATCATGGACGTCAAGAACTTTTTCCTCCGCAAGCGCTCGAGGAAGAGCGAGCTCCCGTTGGCCCCTGTCACTCGCGTAGAGTAA**

**> Coding sequence (CDS) of *PsSCP3***

**ATGGCGCGCGCCTTCTATCGCCTGGGCGCGCTCTGCAGCGGCTCCCCCGTGCTCATGGCGCTGGCGGCGCTGCTCTGCGGCGGCGTGCTGTGCCTGGGGCTGCTCAACATGCGGCTGCAGACCGACCCGCAGGGCCTGTGGGTGCCTCCGCGCAGCGTCGCGGCCCGGGAGCAGGCGCGCTTCGACGAGCTCTTCGGGCCCTTCTTCCGCGTCCAGCAGCTCATCTTCTACGCGGACTCAGACTCAGATGGTCTCTCTGCGACGTGTGACGCCTCGCGCGACCTGGTGCAGCGCCGCTTCCTGCTGCAGATGGCCAAGGTGCAGGCGCAGATCGCCGACGCCGCCGTCACAGTGCAGGGCGACGGCGCCCAGGGCAAGGTGACGCTCTCGCTCGAGGACTTCTGCTACCGCCCCATCCGCGGCAAGGGCTGCCTCGTCACCAGCCCCTTCCAGTACTGGCTCGGCAACGCGTCGCTGCTGGAGGGCGACCCGGACATCAAGCTCACGACCGCGTGCCAGACCACGGACCCACAGCTCCAGGAGCGCGCGCCCTGCATGGACCAGAATGGCGTCCCCGTCATGCGCGACGTCGTGTTCGGCGGCCTGTCGAGGGACGACTGCCACCAGAACCCGGACCCGTGCGGGGAGGCCACGCCGCAGGCCCAGGCGCTCGTGGTCACGTTCCTGCTCAACAACAGGCCCGAGAACGAGACGTACACGAGGTACGTGGAGCAGTGGGAGCAGCAGGCGTTCCTCAAGATTGCAGCACAGGCGGCGGAGGCGCTTAAGCCCTCCTCGACCGCCAACAAGAGCGACGAGTTCATCTGGGACAGCGTGCAGGACCAGGAGCTGGCGGACGTGGGCGTCGACGGCATGCGCCTATCCTACATGGCGGAGCGGTCGGTGGCCGACTCGCTCGTGGTGCAGACCAACCAGAACGCGTTTATTGTGGTGGTGAGCTACCTCGTCATGTTCTTGTACGTGTCGGCGTCGCTCGGCAAGTTCACGGACCCGGTGCGCTCGCGGTTCGGGCTGGGGCTCACGGGCATCCTGATCGTGCTGCTGTCGCTGGGGGCGGCTATGGGGGTGAGCTGCGCCATTCTGCAGATGGAGGTGACGATGATCACGTTGGAGGTCGTGCCGTTCCTGGTGCTGGCTATCGGCGTCGACAACATGTTTATCTTGACCAACGAGTTCGATCGACTCGCGGCTCTGCGTGGCCTCGCAACGCTGGACACCAGGCGCAACACGCGCGACCGGGCAGAGGACGAACTGTTGATGTTGAAGCAGGTGCTGGGAGAAACCATGGTCAATGTCGGACCCTCGATCGTCGTGGCGGCTGTTGCAGAAACGTTGGCGTTCTTGGTGGGGGCTCTCACACGTATCCCCGCCCTAACGAGCTTCTGCGTGGTTGCTGCGCTGGCTGTTGCCGCTGACTTTGCGCTGCAAATGACGTGGTTTGCCTCGGCCCTCGTGTTGGACGCGCGCCGCGTGCGTGCCCGACGCTACGATTTGTTCCCGTGGATGAAGCAGAAACTCACCCTCACGCCGCCCACAAAGGGCAAGCGGAGGATCGAGTCCAAGATTCACTACCAGTATGACTTGCTGGTTGACGAAAGCGAGCGAAGTGACGAGCCAGCAGCTCGTGTGAGCAGCACTGGCACACTTCAGCGTTTCGTGGAGAAAACCTACATCCCGTTCCTGTTACGCCGGTCCACGAAAGTGCTGGTGCTTGTGACGGCTCTTTCAGTGGTAACTCTTAGTGCTTTTGGCTCCTCCGAACTCCCACTCGGACTCGAGCAGGAGCTTGCGGTGCCTACGGATTTTTACCTGCATGAGTACTTCAAGAAACAGACCGCGCTTGGTGAGGCGGGGCCACCAGCTTACGTTGTTCTGGATAGCGATGTGGATTACACAGATGCACGCCTCCAGCAAGATGTGAACGTGCTATTGGATCAGCTTTCCGGTCTTCGCCAGTATATCCAGCTGCCGGTGTGCTCGTGGCTGCATACGTTCAACCAATGGCGTCAAATGCGCTTCTTCCTACAGGACAAGATTAAACAAGGTCAGTGCGATTGTCCGGTGCAGCCAATGGATCCGTTCCCGTACGAACTCGCGAATGTTGGAGTCGAGGACCCCAGTGGAGACGTTGATTTGTTCCTAGCGCCCGAGTATGGGTATGGCGCTCTGGCAACGGCGCATGTGACCCCCAATGCGCTGTTCTACCCGTTGGTGAAGAACTTCACAAAGATCTCAATTGACTCAACGTGCTGCCAGCATTTCGGGCTGTGTGGAGCGCAGTATGAAGGAGACATCATATTCAACGAGCCAAATGCAGGTGATGACGACTCTACGGGCATGTCTATCGTCGGTTCTCGTATACGCTTCCAGCTGAATGCACTCCGCAACCAGTCCATGTTCGTGAACTCGTACTACTACCTTCATGATGTGGTGGGGCGTTGGAGCATCGACCATGCCGCCACTGCCTTCCCGTACGCTCTTGTCTTCGTGTACGAAGAGCAATACACGTACATCCAGGGAGTGGCGCTGCAAAGTGTGCTGCTGGCGCTGGCCGTGGTGTTCGGAGCGCTCTTCGTGCTGATGGACGGGAGCTTGCGGCTGACCACCGTTGTCACCTTGTGCGTGCTGTCGATGACTTTCTCGCAGTTGGGCTTCCTCTTCGTGTGGAATATGATTGCTGGGCCGGGCGCAGAAACGTCGATCAATGCAGTCTCGGTGGTGAATCTGCTCGCCTGTGTTGGGCTTGGTGTCGAGTTTTGCGTGCACACGGCACACCAGTTTGCCTTTTCGCGACGGCATCACCTGGGCACAACTGCCAACGACCACACACGATACGCTCTGAGCAGTGTGGGCGCATCCATCTTCTCCGGTATTACGCTCACCAAGTTCTGCGGCATCGGTGTCCTTGCGTTCGCGCCGTCCATGTTGTTCCGCGTGTACTTCTTCCGCATGTACCTGGGCATTGTCGTGTTGGGCTGCTTCCACGGTCTCGTACTGCTGCCAGTGCTGCTGAGTCTCATTGGCCAGCCGCAGAAGTACCCGAACGATCTCAGCTCGTTTCTACTTTCTGAAGAGCGAGACGACGAGTTAGAGGACGAAGTGAGAGCGTTCAAGTGA**

**> Coding sequence (CDS) of *PsSCP4***

**ATGCGCGTCTCGGCCCTCGCGTCGCTGCTGCTGCCGCTGGGCGTGGCCCGCGCCGCCAGCTCCTCGGGCTCCACGACGTCCACGGCGACCACGACGACAACGGGCACGTCGTCCGTCTCCAGCTACGTGCCCTGGACGCTGGCCGACAACGCCACCAACCTGGCCGACATCAAGGCGCAGCTCACCATGTGCACGTACTCCAAGGTGGAGGAGTGCATCCAGGACCCGGCGCTGGTGCACGAGCTCGGCGCGCTCGTGCGCGCTCCTGGCCACTGCGTGGCCTTCGACTCGTCCTACGTCAACGTCACCACGGCCGGTGTCGCCATCCCCAACCGCTACTACCCGACGTCCGTGGAGGACGCGTACGACGCCGGCTTCTCCAACAAGTTCTCCGAGTGGTCGGACACCAACCGCGAGCAGTTCGAGGTGGACTGCCCGCTGCTCTACAACGAGACCATCGCCCAGGGCGACGACATGCTCTGCTGTACCGAGAACCAGTACACGGGGCTGTCCACGCAGGTGCGCATGATCCCCGGTCTGTGCTCGGCCTGCAAGGAGAACCTGCGCAACATCTTCTGTCAGATGACGTGCAGCCCCAACAACAGCATGTTCCTGGACGTCAACGAGGTGCGCATCATGCCCGGCGACGACGAGCACCCGGACGCCGTGTTCCCCGCCGTGGAGGAGGTCACGTACTACGTGGGCAGCGACTGGATCCGCGACATCTACGACTTCTGCGAGGCCGACAGCTCCTTCTCGCTGCTGTGCAACCCCAATCAGGACTGCCATGACGGCTACGGCCTCCTCGAGTACATGGGCAAGTACGCCTTCAACAGCATCGGGTCTCCGCTCCAGATTAACGTGACGACCATGGACAAGGTCCCCGAGATCAACCAAATGACCGAGTTCTGCCACTGCGACAACGTGAATGCCACCAACTGCATCCTGCCGCAGAACAGCAGGATGACGTCCTGCGTCGGCACCTGCGGCTCGCTGTGTGCCGTGAGCTCGAGCGACGACCGCACGTACACAGAGTCCTGCTACGGCGCCAGCAATGCGGTCGCCACGTCCTCCTCGGCCAGCGGAAGCGTCGGCTCCGGCTCGGACGACTCGACGTGGGCCGAGCTGAACGCGTACCTGGCCAGCAACATCCCGGTGACGGACTGGACGGGCCTCAACTACTTCCTCGTCATCTTCGGTGGAGCCGTCGCCCTGCTGCTCATTGTGGGCTTCATTGTCGCTGGCTGCCGCGAGCGGAGGGCCCGCATCCCCAACCCGCACACGGGCACGCCCCACATCGGCCCGTACACGCCTGAGGCTCACGGCGTGGCGCACGCGATGGAGACGAGCAACACGCGTCTGAGCTATCTGGACGAGCTCATGACGAACAAGCTGCGCACGTGGGCGGTATTCGTGTCGACGGGCAACCGGCCCAAGAAGATGATCCCGATGGTGCTGTGCGTTGTCGCTGTCTGCGTTGTCGGCCTGTACAACATTGATATCGAGACGGACCCGATCAAGCTGTGGGTGTCCTCGTCCAGTACCTCGTACCAGCAGCGCCAGCACTACGGCGAGATCTTCAACCCGTTCTACCGCTCCGAGCAGGTCATTATGGTGCCCAAGGACGGCGGCAACATCTACCGCTCGTCGATCATCAAGGAGGCCATCCGCGTGCAGACTGTGGCAGCCAACGTGACGTACACTTCGGACGACGGTGACGAGACGATTACTCTGGACGACATTTGCTGGAAGGCTACGGGCACGGGCTGCACCGTCAACTCGATCACGCAGTACTTCCAGAACAACATGGAGCACTTCGAGTTCTACGAGAAGTATGGGCTGGAGATGGAGCACTTTAGTAACTGCCTTTACTCTCCGACCACGTCGGACGTGGCGCTTTGCACCGAGTTGAAGAACGCTCTGGAGGATGGCGACTCGCTCCCGTCGAGCATGAGCGACTGCCCCTGCTTGTCAGCGTTTGGCTCGCCCATGAACCTGTACAACACCTACCTGGGTGGCTTCCCTGACGGCGCTGAAAGCAACTACACGTTGTTCCTCGACTCTGTGGCGTTTGTTTCCTCGTACCTGAACTACAACTACGCTGACGACGACAAGAACGAACCTGCCATCAAGTGGGAGCGCGAGTACATCAAGACGATGAAGGAGGAGGCTGCGTCCAACACGATCTTCGACGTCTACTTCTATGCCGAAATCTCCGTCAACGATGAGATCGACGCCGAGTCGAGCAACGGTATGGGCCCTGTGGCGCTCAGTTACTGTTTGATGATCATCTACATCTCGCTCGGTATCAACCGCATCAAGTTCAGCCGCGAGTTTTTCATCTCGTCCAAGATCGTGGCCGGTTTCTGCGGTGTCATGAGCATTGTGTGCGGTGTGGCGTCGACGATCGGTATCTACATGTGGGCTGGAGTCAAGCTCCAGCTTATTATCATGGAAGTGGTTCCCTTCCTGTCGCTGGCCATCGGTGTCGACAACATCTTCTTGATCATCCACGCTATGACGGAGAAGGAAGACCAGATGCGCCGTGAGCAGCCAAGTCTCTTCATCGGTCTGGAGCACAACCCCACGGCGATCGAGGAGATCACCACGACGATTCTGTCCGAGAGTCTGGCGTACATCGGCCCCAGTATCTTCATGGCCTCCGCTGCCGAGTCCGTGGCCTTCGCGTTCGGTTCCATTTCGCCGATGCCTGTCGTGCTGTGGTTCGCCGCCATGGCTTGCTGCGCTGTGGCGATCAACTTCTGCCTCCAGATGACCCTGTTCCTGTCTGTTCTTACGCTGGACAAGCGCCGCGAGCTGAGCGGCAAGTACGACATTATCTTCAAGCGCGCCTCGTACGTCAGGTCGCAGCCGCCTGCCGGTGGCCCGGAGACTCAGCAGACTGCCCAACCCCTTGTCTCTCTCGAGCCCAAGACCCCGGCGCCCGAAGATGCCCGCCGCTCTATCACGCCTGAGAACCGCACGCTGACGGACGTCCTCGACTACTGCGTGGATGTCTACGCGTCGATCCTGACGTACAAGATTGTGAAGCTCGTCGTGCTGCTGGTCTTCCTCTTCTGGACGTTGTGGTCGATCTACTCGATGGAATCGTTGGACCAGGGTCTGCCGCAGAAGGAGGCCATGCCTTCAGACTCGTACATGATTGAGTACTTCAACGCGCTGGATGTGTACCTCGCCACGGGTGTGCCTGTGTACTTCATCGTTGAAACTGGCTACGGTCGCAACCCGGACACGTGGTCGCTGAACGACGAGAGCGTCGAGACGCTCTTCTGCAAGTCGAAGGACATCTGTGGAACGTACTCAATCCCCAACATCATGAACGCGCTGGCCAACCACGGAGACAAGAATGTGACGCACATCAGCCCCGGCACGACCTACTCGTGGATGGATGACTTCTGGGGCTTCGTCAACCCTGACAGCGAGTGTTGCCGTGTGGATTCCGAGGGCGCCTACGTCCCGATCGAAACTGGCAACGACACGTACACGACTCTGCGCGCGGACGACGACACGTGCCTTGCTACGTCGGTGACGATTCCCCCCGTGCCTGAGGCCCAATACATGTCGCTCTTCAGTATGTTCGCGACGGCAAGTGCTGGTACGTCGTGCTCGTACGGTGGTGGTTCGATCTACCGTGGCCAGTTCAGTATCGACTCGGAGCCCATTCCGACCGTCAACGCCTCGACGCCTGCGGTGAAGATCAACAGCAGTGGCTACGGTGACGAGATCACGGCTTGGTCGTACATGGTGACGGGCACGTCGAACCCGACGCAGCAGCGCTACATCGACTCGTACAAGCAGAACCTTGCGGCGGCCGAGTGGATCAGCGAGAAGACTGGTGTGGACATCTGGGTGTACTCGCTGACGTACGTGTACTTCGAGCAGTACCTGACTGTGGTCGACGACGCCTACAAGCTCATCGGTCTGGCGCTGGCGGCTATCTTCGTGATCACGGCGCTGTACCTGGGCAACGTGTTCTACTCGCTGGTGATCGCGTTGACGGCGACCAACATCGTCGTGCTCGTGCTGGGTCTGATGCAGCCGCTGGACATCATGCTCAACGGTCTGTCCATTGTGAACCTGATCATCGCGGCCGGTATCGCCGTCGAGTTCTGCGGTCACTACGTCCGGTTCTTCGCGAAGGCTCGCGGTACGGGCGACGAGCGCGCTCGCGATGCTCTTCGCCAGGTCTTGACGTCGGTTGTGTTTGGCATCACCATCACGAAGGTCATCGGTCTGAGTGTGCTGACACTCGCGGACTCGCGCGTGTTCAAGAAGTACTACTTCCGTATGTACATGATCGTCGTGCTCTGCGGTGTGCTCAACGGCATGCTGCTGCTGCCCGTGCTGCTCAGCACCATCACAGACGTCAAGGACTTCTTCTTGCGTAGAGGATCACGGAAAACCGACCTGCCTTCGGCGCCTGTTACTCGGGTGGAGTAA**
